# STING pathway contributes to the prognosis of hepatocellular carcinoma and identification of prognostic gene signatures correlated to tumor microenvironment

**DOI:** 10.1186/s12935-022-02734-4

**Published:** 2022-10-12

**Authors:** Zhangya Pu, Jinghua Liu, Zelong Liu, Fang Peng, Yuanyuan Zhu, Xiaofang Wang, Jiayan He, Panpan Yi, Xingwang Hu, Xuegong Fan, Jiang Chen

**Affiliations:** 1 Department of Infectious Diseases, Hunan Key Laboratory of Viral Hepatitis, Xiangya Hospital, Central South University, No. 87, Xiangya Rd, Kaifu District, Changsha, 410008 Hunan Province China; 2 Department of General Surgery, Sir Run Run Shaw Hospital, Zhejiang University, Hangzhou, 310000 Zhejiang Province China; 3 Division of Interventional Ultrasound, The First Affiliated Hospital of Sun Yat-Sen University, Guangzhou, Guangdong Province China; 4 Department of Hepatobiliary Surgery, Linyi People’s Hospital, Linyi, Shandong China; 5 NHC Key Laboratory of Cancer Proteomics, Xiangya Hospital, Central South University, Changsha, 41800 Hunan Province China; 6 Department of Infectious Disease, The First Affiliated Hospital, School of Medicine, Zhejiang University, Hangzhou, 310000 Zhejiang Province China

**Keywords:** Hepatocellular carcinoma, Nomogram model, Prognostic gene signature, Immune cell infiltrates, Tumor microenvironment, Immune checkpoints

## Abstract

**Background:**

Hepatocellular carcinoma (HCC) is one of the most malignant solid tumors worldwide. Recent evidence shows that the stimulator of interferon genes (STING) pathway is essential for anti-tumor immunity via inducing the production of downstream inflammatory cytokines. However, its impact on the prognosis and tumor microenvironment of HCC was still limited known.

**Methods:**

We obtained gene expression profiles of HCC from GEO, TCGA, and ICGC databases, and immune-related genes (IRGs) from the ImmPort database. Multivariate Cox regression was performed to identify independent prognostic factors. Nomogram was established to predict survival probability for individual patients. Kaplan–Meier curve was used to evaluate the survival difference. Afterward, ESTIMATE, TISCH, and TIMER databases were combined to assess the immune cell infiltration. Furthermore, the qPCR, western blotting, and immunohistochemistry were done to evaluate gene expression, and in vitro cell models were built to determine cell migratory ability.

**Results:**

We found that gene markers of NLRC3, STING1, TBK1, TRIM21, and XRCC6 within STING pathway were independent prognostic factors in HCC patients. Underlying the finding, a predictive nomogram was constructed in TCGA-training cohort and further validated in TCGA-all and ICGC datasets, showing credible performance. Experimentally, up-regulated TBK1 promotes the ability of HCC cell migration. Next, the survival-related immune-related co-expressed gene signatures (IRCGS) (VAV1, RHOA, and ZC3HAV1) were determined in HCC cohorts and their expression was verified in human HCC cells and clinical samples. Furthermore, survival-related IRCGS was associated with the infiltration of various immune cell subtypes in HCC, the transcriptional expression of prominent immune checkpoints, and immunotherapeutic response.

**Conclusion:**

Collectively, we constructed a novel prognostic nomogram model for predicting the survival probability of individual HCC patients. Moreover, an immune-related prognostic gene signature was determined. Both might function as potential therapeutic targets for HCC treatment in the future.

**Supplementary Information:**

The online version contains supplementary material available at 10.1186/s12935-022-02734-4.

## Background

Hepatocellular carcinoma (HCC) is the major histological type of liver cancer, accounting for 70% to 90%. It is ranked the second leading cause of cancer-related mortality among all solid tumors worldwide. In the past decades, the epidemiological trend of HCC is changed gradually, with increasing incidence in most regions in the world and decreased ratio in some countries in ascian [1, 2]. The initiation and progression of HCC are demonstrated to be associated with chronic liver diseases, such as hepatitis B virus (HBV) and hepatitis C virus (HCV) infection, cirrhosis formation, and exposure to toxic substances, such as aflatoxin B1, and alcohol consumption et al. [3, 4].

Currently, surgical therapies are thought of as the curative treatment for HCC patients who are diagnosed at an early stage, especially for the population with single lesions and well-preserved liver function. However, the recurrent ratio after five years of surgery among patients was high at up to 70%, which limited the clinical benefits [2, 5]. Then, locoregional therapies such as radiofrequency ablation (RFA) and transarterial chemoembolization (TACE) were the alternative therapeutic strategies to reduce surgical recurrence. When considering the therapeutic approaches for HCC patients at more advanced stages, it was widely accepted that HCC is not sensitive to conventional systemic anti-cancer treatment. The realization facilitated the pace of research for more tailored treatments, such as significant pathways correlated to tumor progression and prognosis, and crucial biomarkers including sorafenib (multi-kinase inhibitors) and lenvatinib (tyrosine kinase inhibitors). Although the advancement of diagnosis and therapeutic regimens has improved the prognosis of HCC patients to some extent, the overall survival at 5 years is still unsatisfactory [2, 6, 7].

In the past decade, immunotherapies, especially targeting the prominent immune checkpoints like anti-programmed cell death protein 1 (anti-PD-1) and anti-CTLA-4, have attracted growing attention since they showed unprecedented efficacy in a variety of cancer types, such as melanoma, breast cancer, lung cancer, and liver cancer [8, 9].

Now, several anti-PD-1 antibodies have been approved by the Food and Drug Administration (FDA) such as nivolumab which was permitted to treat patients with advanced HCC for the second-line approach, and pembrolizumab which was also favored to use in the patients diagnosed at advanced stages [9, 10]. Moreover, a recent clinical trial targeting unresectable HCC composed of atezolizumab (anti-PD-L1) and bevacizumab (anti-VEGF) reported that increased overall survival (OS) and progression-free survival (PFS) were observed in the combined group than sorafenib alone group [11]. Whereas nearly 80% of patients with HCC still didn’t show an ideal response to immunotherapies [6, 12]. Thus, it is imperative to comprehensively understand the tumor microenvironment aiming to uncover more valuable biomarkers associated with predicting response to immunotherapeutic treatments.

It is known that DNA could stimulate innate immunity when recognized by pattern recognition receptors (PRRs), then inducing the initiation of inflammatory production, secretion of interferons (IFNs) as well as cytokines. Subsequently, the adaptive immune was activated [13, 14]. Cancer treatments such as radiotherapy, chemotherapy, and molecular targeted drugs could largely increase DNA damage, which, to some extent, stimulates the immune system and enhances the anti-cancer immune response. The cytosolic DNA is a dangerous signal that could be recognized by cyclic GMP-AMP synthase (cGAS), a sequence-independent but length-dependent manner. Then the stimulator of interferon genes (STING) pathway was activated and further mediated the recruitment and activation of the downstream biomarkers like TANK-binding kinase 1 (TBK1) and interferon regulatory factor 3 (IRF3) et al. [13, 15, 16]. Recent increasing evidence showed that the activation of STING pathway was significantly associated with anti-tumor responses such as non-small cell lung Cancer (NSCLC), small cell lung cancer (SCLC), pancreatic ductal adenocarcinoma (PDACs), and colorectal cancer (CRC) [13, 17–21]. Nevertheless, our knowledge concerning the STING pathway in expression difference between tumors and adjacent normal tissues, biological functions, and its potential correlation to prognosis in various cancer types including HCC remains obscure in a large part.

In the current study, we comprehensively explore the prognostic value of STING pathway in HCC cohorts obtained from The Cancer Genome Atlas (TCGA, n = 366), Gene Expression Omnibus (GEO, GSE14520, n = 242), and International Cancer Genome Consortium (ICGC, n = 243). Then, five survival-related biomarkers within STING signal panel, namely NLRC3, STING1, TBK1, TRIM21, and XRCC6, were determined via multivariate Cox regression analysis and further used to establish a prognostic nomogram model for predicting the survival probability of individual HCC patients. Simultaneously, TBK1 was experimentally demonstrated to enhance the migratory ability of HCC cells using in vitro cell models. Subsequently, three prognostic biomarkers of VAV1, RHOA, and ZC3HAV1 within immune-related co-expressed gene signatures (IRCGS) were identified after an integrated analysis of co-expressed genes of the fiver survival-related members of STING pathway. Furthermore, we found that three survival-related IRCGS were correlated to immune cell infiltrating and immunotherapeutic response. All these findings are prone to profoundly understanding the prognostic value of STING pathway in HCC, and give us clues to ascertain potential biomarkers for future immunotherapeutic treatment.

## Methods

### Data acquisition

The gene-expressed profile and clinical information of HCC samples were obtained from The Cancer Genome Atlas (TCGA) (https://portal.gdc.cancer.gov/), International Cancer Genome Consortium (ICGC) https://dcc.icgc.org/, and Gene Expression Omnibus (GEO) (https://www.ncbi.nlm.nih.gov/gds/), which was named as TCGA (n = 366), ICGC (n = 243), GSE14520 (n = 242), and GSE84005 (n = 38, without clinical traits), respectively. Correspondingly, the adjacent normal liver tissues included 50 cases for TCGA, 202 cases for ICGC, 241 cases for GSE14520, and 38 cases for GSE84005. The TCGA cohort was divided into TCGA-training (n = 183) and TCGA-test (n = 183) datasets at the wide accepted cutoff of 50% underlying randomized rule. Additionally, the entire ICGC and GSE14520 cohorts were used as the external testing sets. The demographic and clinical traits of HCC patients in various datasets were summarized (Additional file 1: Table S1).

Furthermore, sixteen markers of the Rectome STING pathway composed of CGAS, DDX41, DTX4, IFI16, IRF3, MRE11, NLRC3, NLRP4, PRKDC, STAT6, STING1, TBK1, TREX1, TRIM21, XRCC5, and XRCC6 were downloaded from the GSEA Molecular Signatures Gene Set Database (MSigDB) v7.1. All immune-related genes (IRGs) of 1793 markers were downloaded from the ImmPort database (https://www.immport.org/shared/home). The UALCAN online tool (http://ualcan.path.uab.edu/) was operated to explore co-expressed genes via Pearson’s correlation coefficient and EMTome (http://www.emtome.org/) was used to retrieve gene markers related to epithelial-mesenchymal transformation (EMT). Meanwhile, the clustered regularly interspaced short palindromic repeats (CRISPR) data of genes in various cell lines were retrieved from DepMap portal that was open access for researchers to discover markers related to cancer vulnerabilities (https://depmap.org/portal/). A lower score means a more likelihood that the gene of interest is essential in a specific cell line. Correspondingly, -1 is equal to the median of all essential genes.

### Multivariate Cox regression analysis and risk score calculation

To evaluate the correlation of gene expression and survival probability in HCC patients, multivariate Cox proportional hazard regression analysis was operated using R package “survival” and the package of “forestplot” was used to visualize the results. Next, the risk score of individual patients was calculated based on the following formula: risk score = (expressed value of gene 1 × coefficient) + (expressed value of gene 2 × coefficient) + … + (expressed value of gene n × coefficient). The coefficient was the variance in the expressed level of a specific gene in all samples. The patients in a certain HCC dataset were further categorized into low-risk and high-risk groups using the best cutoff value via X-tile software. Moreover, the relationship between risk score stratification and clinicopathologic factors of HCC patients was evaluated by independent t-tests.

### Establishment and evaluation of predictive nomogram model

In order to provide a quantitative analysis tool to foretell the survival probability for each HCC patient, the predictive nomogram was established on the basis of survival-related biomarkers within STING pathway. Meanwhile, the calibration curves at the indicated timepoint of 1-, 3-, and 5-year survival were measured to evaluate the consistency between predictive and practical survival ratios. Similarly, the area under the time-dependent receiver operating characteristic (ROC) curve (AUC) was constructed to assess the performance of the nomogram. The reliability was thought of as low, moderate, and high with the AUC value of < 0.5, 0.5–0.7, and > 0.7, separately.

The process was analyzed using R packages of “rms”, “survival”, and “timeROC”.

### Identification of molecular clusters and functional analysis

The overlapped gene signature of co-expressed genes of each survival-related marker among the STING panel was determined by the online Venn tool (http://jvenn.toulouse.inra.fr/app/example.html). The molecular complex detection (MCODE) plugin included in Cytoscape software (version 3.8.2) was performed to identify the key molecular clusters. The ClueGO algorithm was further explored to understand the biological functions of genes included in the key modules and their correlation to the immune process. The immune-related co-expressed gene signature (IRCGS) included in the key clusters was ascertained after intersecting with all IRGs.

### Estimation of immune cell infiltration and immunotherapeutic response

TIMER 2.0 is a comprehensively public resource for evaluating the infiltration of immune cell subtypes across diverse cancer types from TCGA and has been widely used in immune-related research [22]. The correlation of IRCGS and six classical immune subtypes of B cells, CD4^+^ T cells, CD8^+^ T cells, macrophages, neutrophils, and dendritic cells (DC) was explored in TIMER 2.0. Similarly, ESTIMATE was performed to calculate immune-related scores including stromal, immune, and ESTIMATE scores to reflect the profile of immune cell infiltration within the tumor microenvironment on the basis of the expression of specific genes via R package of “ESTIMATE” [23]. Then, the R package of “CIBERSORT” was executed to count the fraction of 22 types of immune cells in the HCC cohort from TCGA [24]. What’s more, The Tumor Immun Single-cell Hub (TISCH) database (http://tisch.comp-genomics.org/home/), a popular open-access database focused on the tumor microenvironment underlying single-cell sequence, was used to further estimate the correlation of biomarker expression and the infiltration of various immune cell types at the single-cell level. Additionally, the Tumor Immune Dysfunction and Exclusion (TIDE) database (http://tide.dfci.harvard.edu/) was operated to estimate the influence of biomarkers on the immunotherapeutic response in various cancer types.

### Cell culture and transfection

Human HCC cell lines including HepG2, HA22T, MHCC-LM3, Huh7, JHH-7, and HLF were purchased from the American Type Culture Collection (ATCC, Manassas, VA, USA). The human immortalized liver cell line of L02 was bought from the China Center for Type Culture Collection (CCTCC, China). Dulbecco’s modified Eagle’s medium (DMEM, Gibco, USA) was used to culture HepG2, MHCC-LM3, Huh7, JHH-7, and HLF cells. Meanwhile, L02 and HA22T cells were cultured in RPMI-1640 medium (Gibco, USA). All mediums used in the study contained 10% fetal bovine serum (FBS, Gibco, USA) and 1% penicillin/streptomycin (Beyotime, China). Then, the cells were incubated at a constant temperature and humidity incubator at 37 °C with 5% CO_2_.

Small interfering RNA (siRNA) targeting TBK1 designed by Hanbio Biotechnology, Shanghai, China, and exogenous TBK1 plasmid purchased from Bochu Biotechnology, Changsha, China, were transfected into cells using Lipofectamine 3000. TBK1-siRNA target sequences (5′–3′) #1: AAGGUACUGGCAAUUCUGCTT. #2: AUUGUUCCCUGAGAACUGGTT.

### RAN extraction and real-time quantitative PCR

Total RNA was collected from cells using Trizol reagent (Thermo Fisher, USA) following the manufacturer’s instructions. The quality and concentration of RNA were evaluated by the NanoDrop ™ 1000 Spectrophotometer (Thermo Fisher, USA) with the acceptance of OD260/OD280 ratios of 1.8–2.0, and the OD260/230 ratios of 2.0–2.2. Then, the reverse transcription kit (PrimeScript RT reagent Kit, Takara, Japan) was used to synthesize the complementary DNA and SYBR green Master Mix (Thermo Fisher, USA) was performed to amplify DNA. Then, GAPDH was regarded as the normalizer for RNA quantification. The primers of real-time quantitative PCR (qPCR) for gene markers of the STING pathway were presented (Additional file 2: Table S2).

### Western blot

Cells were collected and lysed by lysis RIPA buffer that was pre-cooled on the ice and contained the complete protease and phosphatase cocktail. Then, cell lysates were centrifuged at 4 °C, 12,000 g for 20 min to remove cell debris. The BCA protein assay kit (Thermo Scientific, USA) was used to measure protein concentration and all samples were further diluted into the same concentration with an equal volume. The western blotting (WB) was conducted as previously described [25]. Briefly, protein samples were separated underlying the 10%-15% sodium dodecyl sulphate–polyacrylamide gel electrophoresis (SDS-PAGE) gel followed by being transferred to the polyvinylidene fluoride (PVDF) membrane. The primary antibodies were used to incubate target proteins at 4 ℃ overnight, which included VAV1 (Proteintech, China, Cat# 16364-1-AP), RHOA (Proteintech, Cat# 66733-1-Ig), ZC3HAV1 (Protein, Cat # 16820-1-AP), TBK1 (Proteintech, Cat # 28397-1-AP), Flag-tag (Affinity, China, cat # T0053), GAPDH (Proteintech, Cat#60004-1-Ig), and α-tubulin (Proteintech, Cat# 66031-1-Ig). Then, the target bands were incubated with the corresponding anti-mouse or anti-rabbit secondary antibody linked with horseradish peroxidase (HRP) (Fushen, China, Cat# FSM0075and Cat #FSM0056,) and visualized by enhanced chemiluminescence (MilliporeSigma, USA). The Image J software was operated to determine the relative protein quantification.

### Immunohistochemistry

The study was approved by the Institutional Ethics Committee of Linyi People’s Hospital, Linyi, Shandong Province, China. Eight HCC samples and the adjacent liver tissues with formalin-fixed and paraffin-embedded were collected with informed consent. Immunohistochemistry (IHC) was performed as previously presented [26]. Briefly, the sample slides from HCC patients have experienced the sequential steps of dewaxing, rehydration, antigen-retrieval, permeabilization, and blocking before hybridization with primary antibodies of VAV1, RHOA, and ZC3HAV1 at 4 °C overnight, the catalog number was the same as mentioned above. Then, all samples were incubated with biotinylated goat anti-rabbit/mouse immunoglobulin at room temperature for one hour followed by visualization using diaminobenzidine (DAB) (GK500705 kit, DAKO, China).

To qualify the protein expressed level of indicated genes, five representative fields in each sample slide were randomized photographed, and assessed by two experienced pathologists. The staining intensity was defined as negative, weak, low positive, positive, and strong positive with scores of 0, 1, 2, and 3 points, respectively. The assessment process was operated in Image J software with the IHC Profiler plug-in.

### Wound-healing and migration assay

For the wound-healing assay, 1 × 10^6^ cells were cultured in 6-well plates and scratched with a 10 μL sterilized pipet tip when the cell confluence is about 90%, followed by gently rinsed with 1 × PBS. Then, cells were cultured for another 24 h in a serum-free medium. Images were acquired under a microscope at a specific time point. For the migration assay using a transwell system, 1 × 10^5^ cells were suspended in 200 µL of serum-free DMEM medium and plated in the upper compartment of 24-well Transwell chambers (8 μm size, Corning, USA, Cat# 3422,). The lower chamber was filled with the chemotactic medium containing 10% FBS. The cells that couldn’t penetrate the inserts after incubation of 24–48 h were removed. And the inserts were stained with crystal violet staining Solution (Beyotime, China, Cat #C0121,) after fixation of 4% paraformaldehyde. Then, the inverted microscope was used to count the migratory cells.

### Statistical analysis

Cox regression analysis was conducted via R (version 4.0) package “survival”, along with hazard ratio (HR) and 95% confidence interval (CI). For continuous variables, a student t-test with the one-tailed method was used to compare two groups, and the Brown-Forsythe test underlying one-way ANOVA for comparing three or more groups. The Chi-squared test or Fisher’s exact test was performed to compare categorical variables. Moreover, the Kaplan–Meier (K-M) curve with a Log-rank test was operated to evaluate survival differences in various groups. Pearson’s correlation coefficient was adopted for evaluating the correlation of two variables. The *P* value of < 0.05 was considered statistical significance. All *P < 0.05, **P < 0.01, ***P < 0.001, ****P < 0.0001, and ns indicated no statistical significance.

## Results

### Identification of survival-related markers within STING pathways in HCC patients

**Fig. 1:**
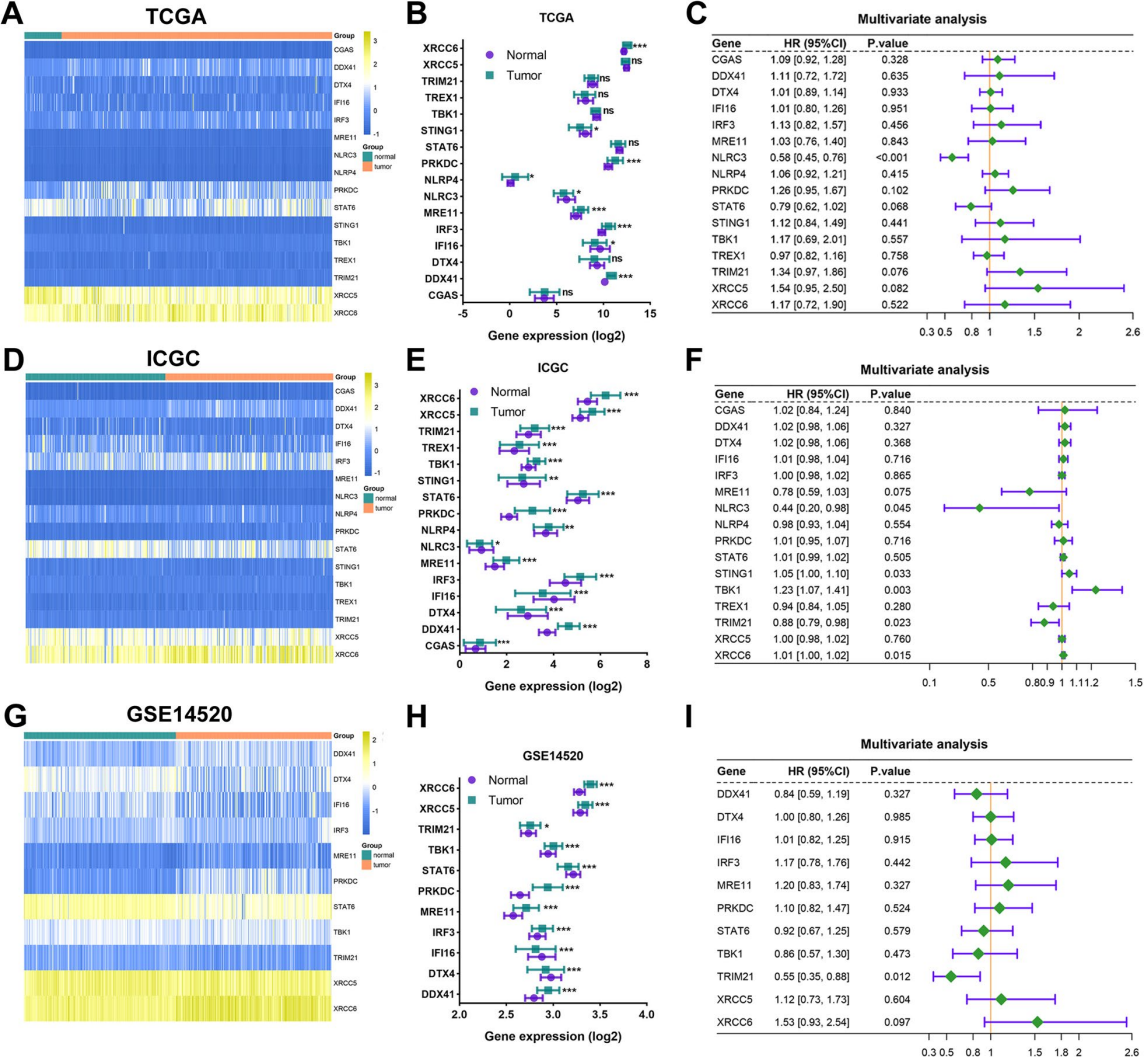
The transcriptional expression profile of STING pathway and its correlation to OS in patients with HCC from diverse datasets. **A, D, and G** The heatmaps showed the transcriptional expression profile of markers within STING pathway in tumor and normal tissues of individual HCC patients in TCGA, ICGC, and GSE14520 cohorts, respectively. **B**, **E**, and **H** The expressed difference of markers in the tumor and normal group was further summarized. **C, F, and I** Multivariate Cox regression analysis was executed to evaluate the correlation of members of STING pathway and OS in TCGA, ICGC, and GSE14520 HCC datasets, respectively. OS: overall survival. All *P < 0.05, **P < 0.01, ***P < 0.001, ns: no significance

**Fig. 2:**
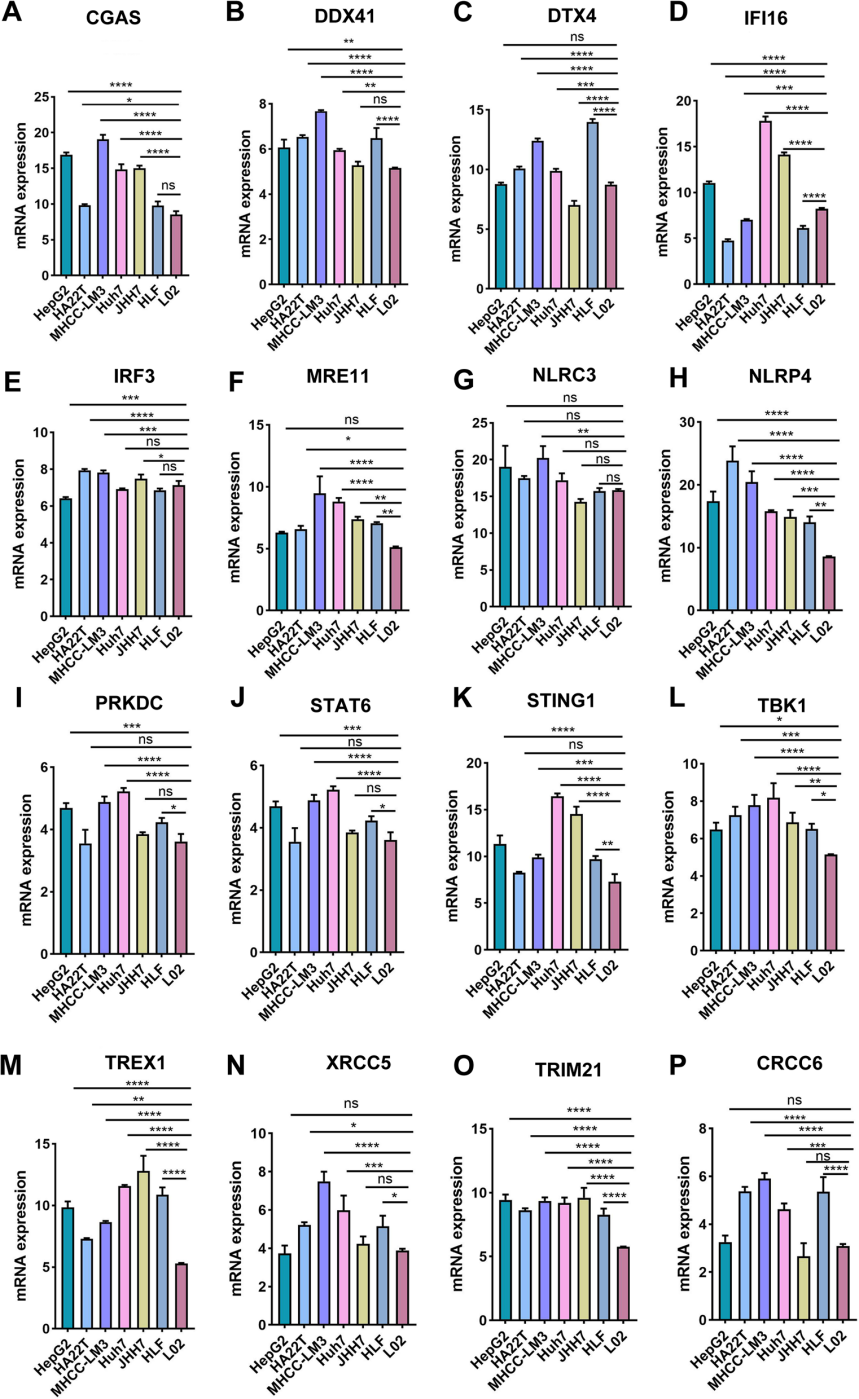
The qPCR assay was performed to determine the transcriptional expression of members in STING signal path in human HCC cells and immortalized liver cells. All *P < 0.05, **P < 0.01, ***P < 0.001, ****P < 0.0001, ns: no significance

We at first presented the transcriptional expression profile of gene markers within the STING pathway in various HCC datasets from TCGA (n = 366), ICGC (n = 243), and GSE14520 (n = 242), among which GSE14520 dataset underlying chip platform only showed the transcriptional data of eleven members included in the STING pathway (Fig. 1A, D, and G). Then, the expressed difference of each marker between the patients in the HCC and normal groups was compared. In ICGC and GSE14520 datasets, it was indicated that the genes of XRCC6, XRCC5, TRIM21, TREX1, TBK1, STING1, STATE6, PRKDC, NRLP4, NLRC3, MRE11, IRF3, DDX41, and cGAS showed higher expression in HCC than the adjacent normal liver group. Reversely, the expression of IFI16 and DTX4 was increased in normal liver tissues than in HCC samples. In the HCC cohort from TCGA database, the expression of XRCC5, TRIM21, TREX1, TBK1, STAT6, DTX4, and cGAS didn’t demonstrate a significant difference between HCC and normal groups. Consistently, the up-regulated expression of IFI16 was observed in the normal group (Fig. 1B, E, and H). Next, the qPCR assay was operated to detect the transcriptional expression of sixteen gene members included in the STING pathway in various human HCC cell lines and immortalized normal liver cells. We found that most genes except for NLRC3 indicated an increased mRNA expression in HCC cells than in normal liver cells. Meanwhile, the difference in the expressed tendency of a specific gene in a variety of HCC cells indicated heterogeneity (Fig. 2 A–P).

Next, the relationship between the expression of gene markers in the STING pathway and survival probability in HCC patients was explored via multivariable Cox regression analysis (Additional file 3: Table S3). The result showed that the gene markers of NLRC3 (P < 0.001 in TCGA and P = 0.045 in ICGC database), TRIM21 (P = 0.012 in GSE14520 and P = 0.023 in ICGC cohort), and other three genes included in the ICGC cohort, namely STING1, TBK1, and XRCC6, presented a significant association with prognosis in HCC patients (Fig. 1C, F, and I).

### Construction and verification of the prognostic nomogram in HCC cohorts underlying survival-related STING signaling markers

**Fig. 3:**
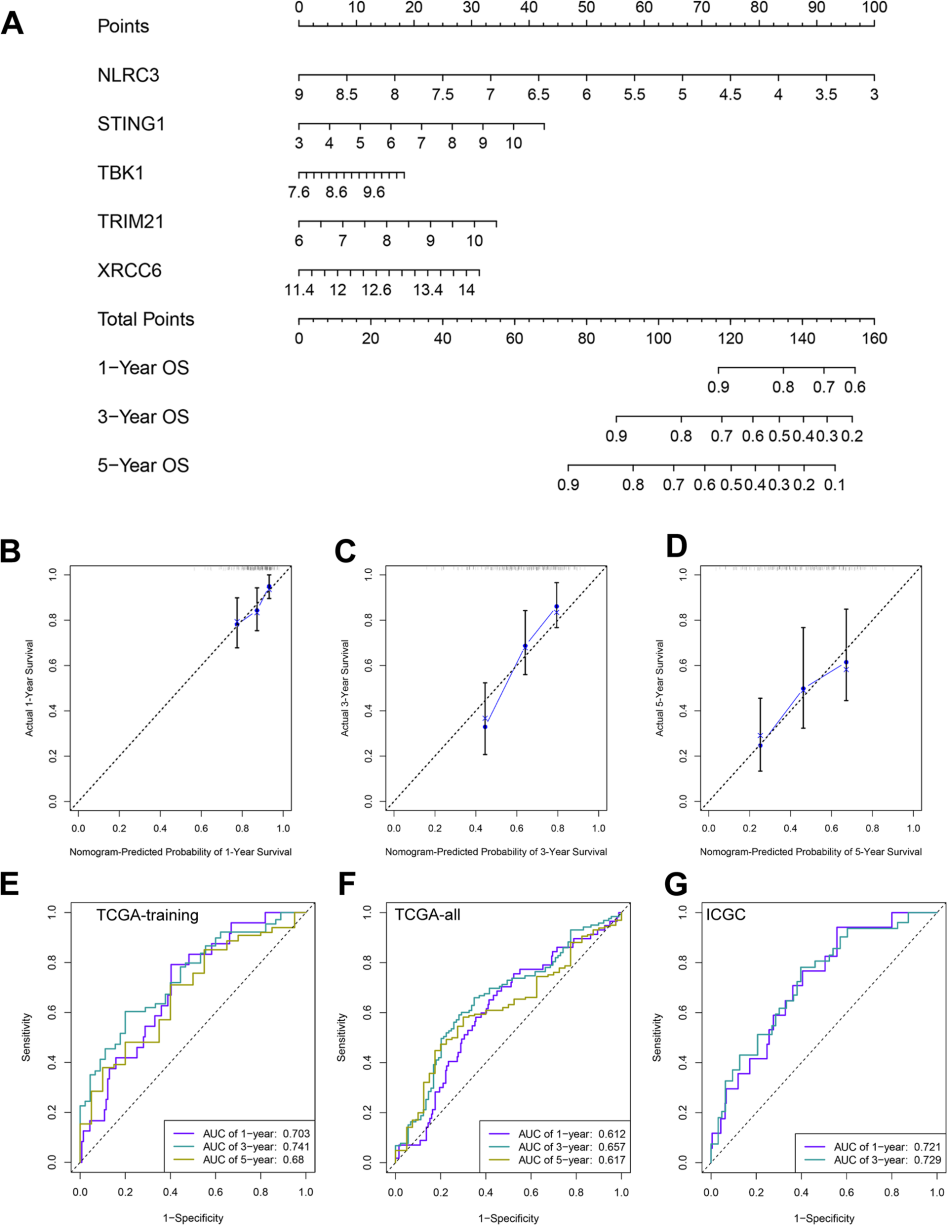
Establishment of clinical prognostic nomogram and evaluation of its performance in HCC patients underlying five survival-related markers of STING pathway. **A** Five survival-related biomarkers including NLRC3, STING1, TBK1, TRIM21, and XRCC6, were utilized to build a predictive nomogram using the TCGA-training cohort. **B–D** The calibration curve was drawn to assess the consistency between nomogram-predicted survival probability and actual survival time at 1-year, 3-year, and 5-year OS in TCGA-training cohort, separately. **E–G** The area under ROC was further calculated to verify the prognostic performance of nomogram in HCC patients from TCGA-training, TCGA-all, and ICGC cohorts. ROC: Time-dependent receive operating curve

According to five survival-related gene markers within STING pathway including NLRC3, STING1, TBK1, TRIM21, and XRCC6, we constructed a predictive nomogram using the TCGA-training cohort, aiming to provide a quantitative analysis tool to evaluate the survival risk at 1-, 3-, and 5-year for individual patients with HCC (Fig. 3A). Subsequently, the calibration curves indicated the consistency between predictive survival possibility at 1-, 3- and 5-year of OS and the actual probability in the TCGA-training cohort and internal TCGA-all and external ICGC validated cohorts (Fig. 3B–D, Additional file 4: Figure S1). More importantly, The area under the receiver operating characteristic (ROC) curve at 1-, 3-, and 5-year overall survival (OS) for HCC patients in the TCGA-training dataset was drawn, which indicated excellent credibility with the ratio of 0.703, 0.741, and 0.680, respectively, and further was validated in the test cohorts including TCGA-all and ICGC datasets (Fig. 3E–G). These results demonstrated the convincing performance of the predictive nomogram. Additionally, the co-expressed correlation between the five gene markers included in the nomogram was evaluated in the TCGA-training, TCGA-all, and ICGC cohorts (Additional file 5: Figure S2).

### Relationship between risk score calculated by survival-related markers of STING pathway and the progression of HCC patients

**Fig. 4:**
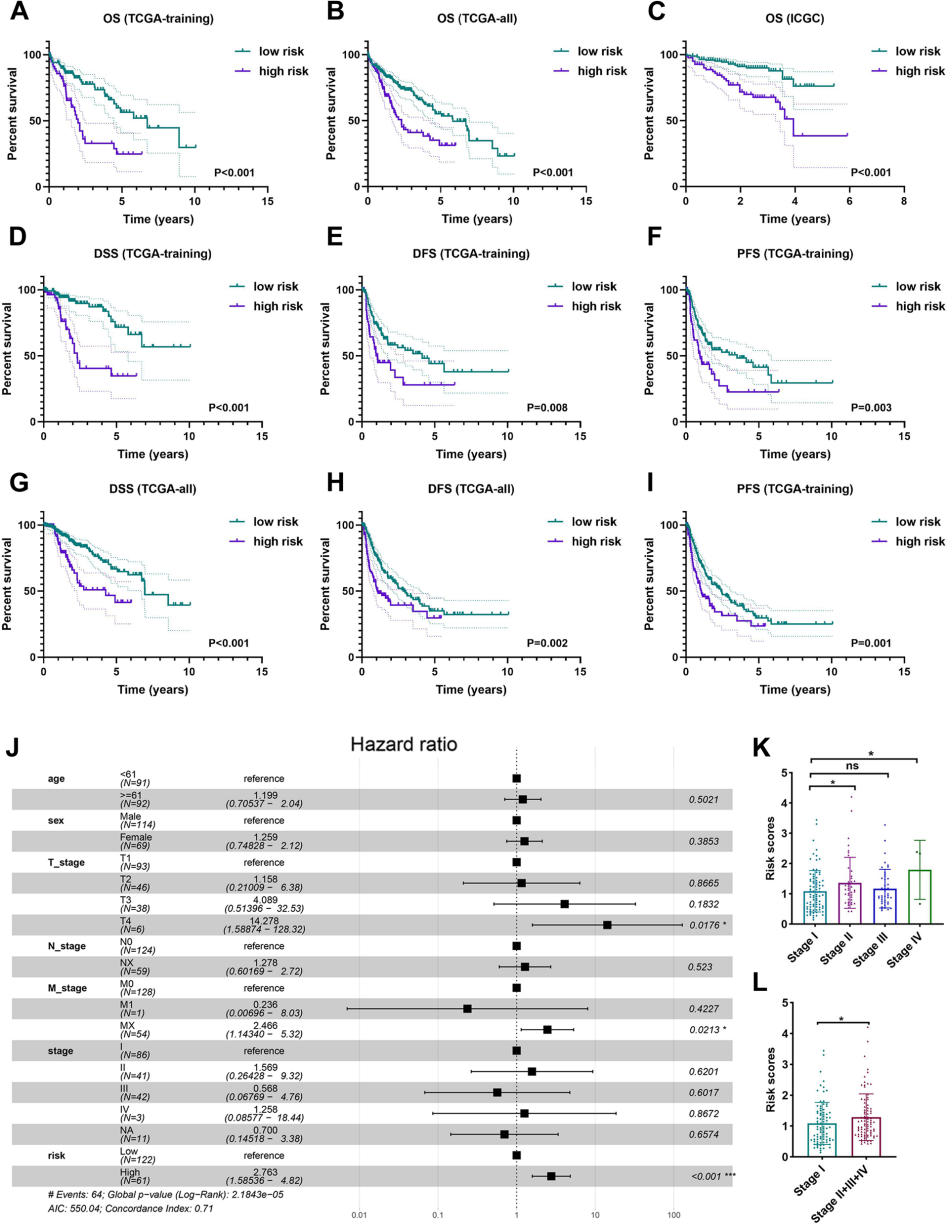
Comprehensively understand the value of risk score calculated by five survival-related markers within the STING pathway in disease progression and prognosis of HCC patients. **A–I** The patients in the high-risk group experienced a shorter survival probability including OS (**A–C**), DSS (**D** and **G**), DFS (**E** and **H**), and PFS (**F** and **I**) than patients in the low-risk group from various HCC cohorts. **J, K, and L** The multivariate Cox regression analysis was constructed in TCGA-training dataset to demonstrate that risk score was an independent risk factor of survival probability (**J**) and significantly associated with the disease progression (**K** and **L**). OS: overall survival. DSS: disease-specific survival. DFS: disease-free survival. PFS: progression-free survival. All *P < 0.05, ***P < 0.001, ns: no significance

The risk score for each HCC patient in the TCGA-training, TCGA-all, and ICGC dataset was counted according to the coefficient obtained from multivariate Cox regression analysis. The patients from the TCGA-training cohort were divided into low- and high-risk groups using the X-title software to determine the best cutoff value of risk score (Additional file 6: Figure S3). Both in the TCGA-training cohort and validated cohorts of TCGA-all and ICGC, the patients included in the high-risk group experienced a poor survival probability than patients within the low-risk group (Fig. 4A–C). Consistently, a superior ratio of disease-specific survival (DSS), disease-free survival (DFS), and progression-free survival (PFS) were observed in the patients within the low-risk groups than those in the high-risk group in the TCGA-training (Fig. 4D–F) and TCGA-all cohorts (Fig. 4G–I).

To explore whether the risk score was an independent indicator of prognosis, the clinical characteristics including age, sex, T stage, N stage, M stage, and AJCC stage combined with the risk score were further synthetical analyzed using multivariate Cox regression in the TCGA-training cohort. As shown in Fig. 4J**,** the risk score factor demonstrated its independent value of predictive prognosis in HCC patients. Moreover, the relationship between the distribution of risk scores and multiple clinicopathologic characteristics was evaluated via conducting independent t-tests. In the TCGA-training cohort, the risk score was significantly higher in patients at advanced stages (Fig. 4K, L). Similarly, we obtained a consistent tendency in the TCGA-all (Additional file 7: Figure S4A-C) and ICGC datasets (Additional file 7: Figure S4D-F).

### Up-regulation of TBK1 promoted the migratory ability of HCC cells

**Fig. 5:**
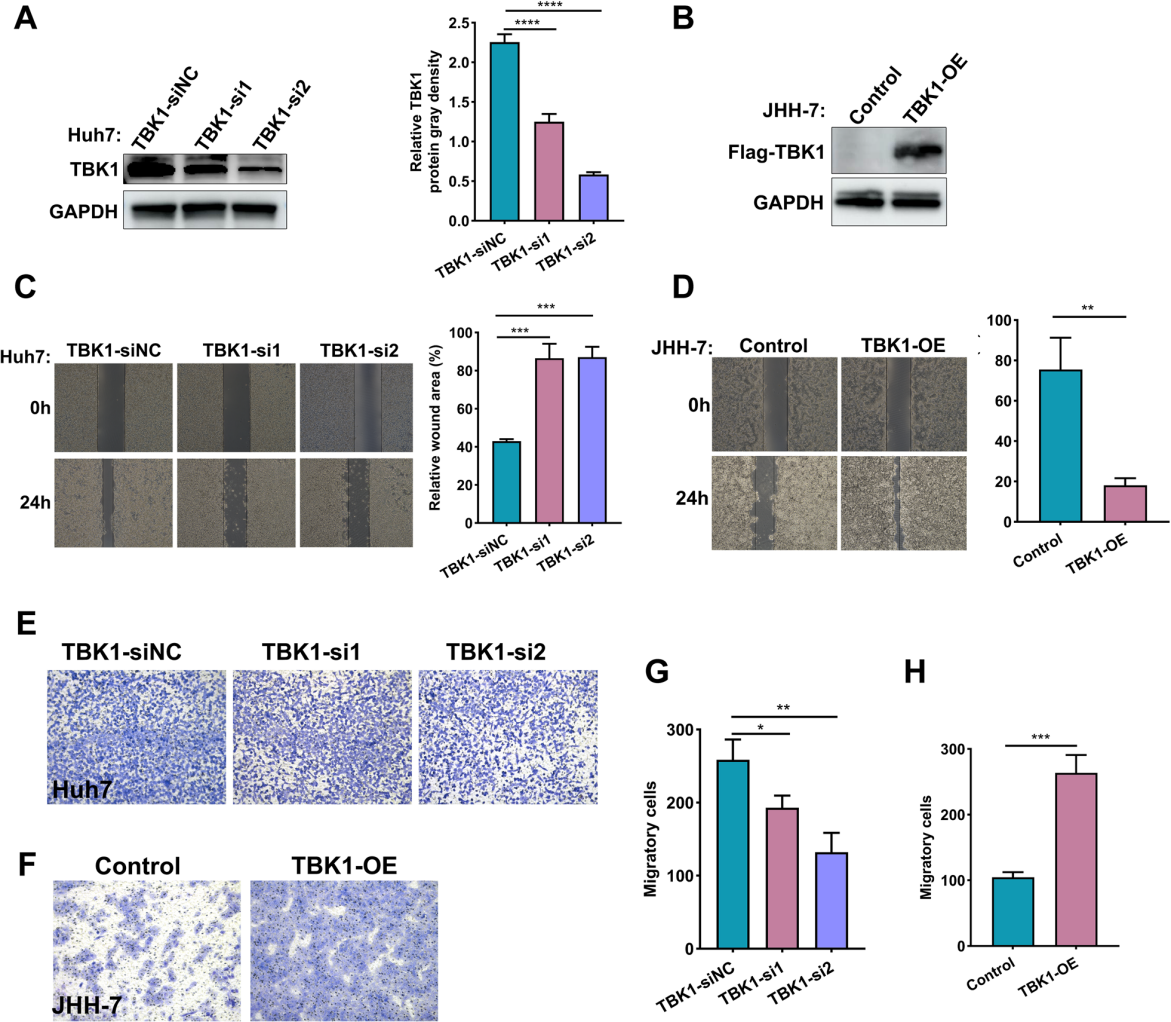
TBK1 is significantly associated with migratory probability in human HCC cells. **A, B** Western blotting was used to verify the exogenously depleted and overexpressed efficacy of TBK1 in HCC cells, respectively. **C, D** The wound healing assay was performed to evaluate the difference in migratory ability of HCC cells with specific treatment. **E–H** The transwell system was further done to determine the migratory variation of HCC cells in specific experimental conditions. All *P < 0.05, **P < 0.01, ***P < 0.001, ****P < 0.0001

The GSE84005 dataset was regarded as another externally validated HCC cohort for detecting the expression of five survival-related STING pathway markers, it was noticed that the expression of NLRC3 and TBK1 was elevated in the tumor group, showing a consistent expression trend with the observation in HCC cohorts from TCGA, ICGC, and GSE14520 datasets (Additional file 8: Figure S5). Afterward, the impact of TBK1 expression on the migratory ability of HCC cells was investigated since its exogenous plasmid is easy to be transfected into HCC cells. The western blotting was performed to monitor its transfected efficacy (Fig. 5A, B). The wound-healing assay displayed that the up-regulated expression of TBK1 enhanced the migratory probability of HCC cells, reversely, the phenomenon was attenuated in HCC cells with TBK1 depletion (Fig. 5C, D). Furthermore, the transwell system was operated again to assess the influence of TBK1 expression on HCC cell migration, we obtained a consistent result (Fig. 5E–H).

### Determination of immune-related co-expressed gene signatures (IRCGS) underlying integrated analysis of survival-related STING pathway markers

**Fig. 6:**
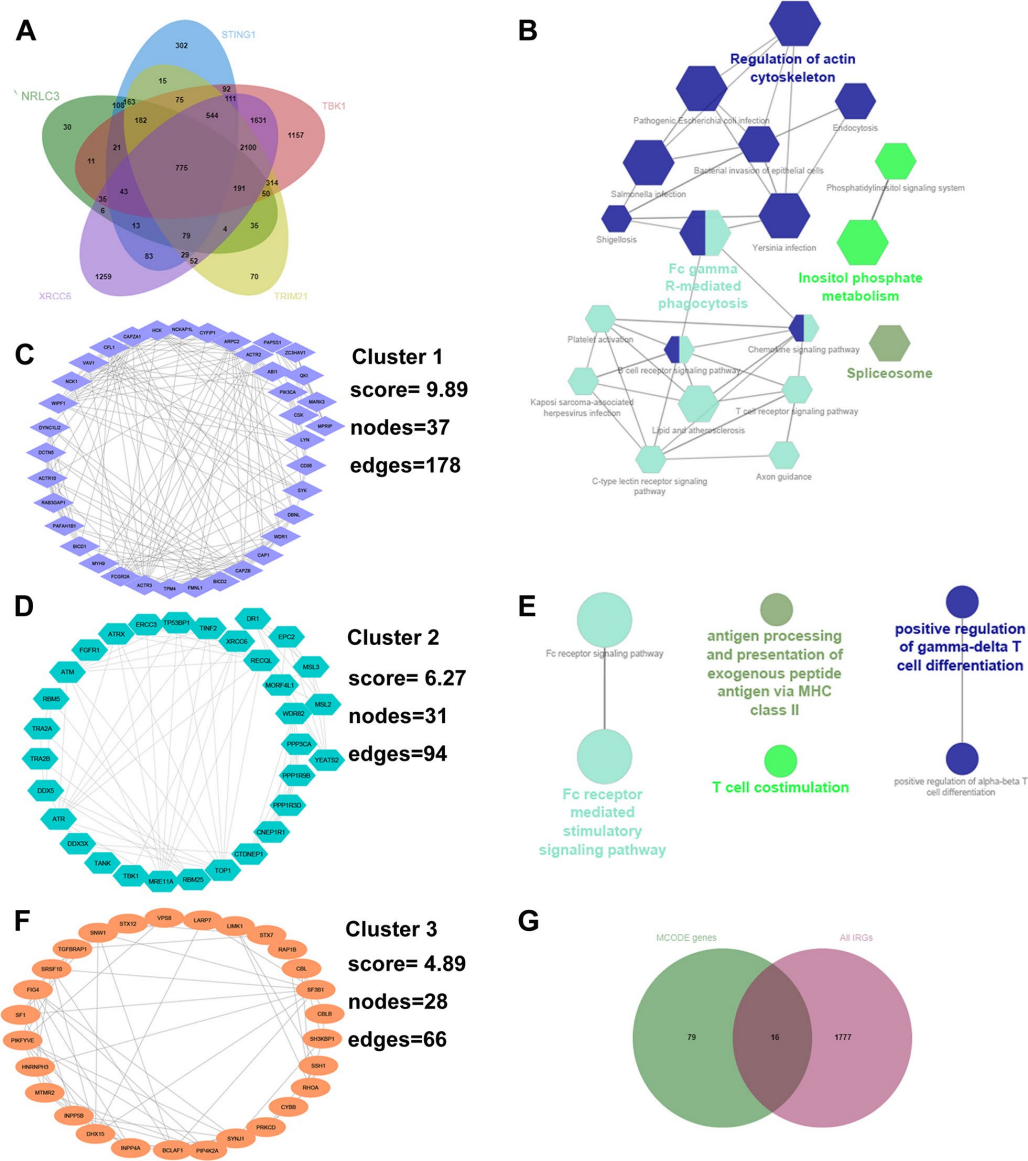
Identification of co-expressed gene signatures of five survival-related markers included in the risk score model and further analysis of their biological functions.** A** Venn diagram showed that a total of 775 overlapped co-expressed genes were screened based on Pearson’s correlation coefficient from UALCAN database. **C, D, and F** MCODE algorithm in Cytoscape software (version 3.8.2) was operated to identify three crucial clusters for overlapped co-expression genes. **B and E** The enriched molecular pathways based on KEGG database and the involved immune processes were performed via the ClueGo program. **G** All immune-related genes were downloaded from ImmPort database and further intersected with co-expressed gene signatures within three crucial clusters to distinguish the immune-related co-expressed gene signatures (IRCGS). MCODE: molecular complexes detection. KEGG: Kyoto Encyclopedia of Genes and Genomes

We adopted the UALCAN online tool underlying Pearson’s correlation coefficient to acquire the co-expressed genes of five survival-related STING pathway markers, namely NRLC3, STING1, TBK1, TRIM21, and XRCC6, in the HCC cohort from TCGA database, among which the top 20 co-expressed genes for each survival-related STING pathway marker were summarized (Additional file 9: Figure S6A-E). Sequentially, the five batches of co-expressed genes were intersected with each other to obtain 775 overlapping co-expressed gene signatures (Fig. 6A, Additional file 10: Table S4). And the KEGG pathway and immune-related processes were investigated in order to understand their potential biological functions (Additional file 9: Figure S6F-G). MCODE, a novel theoretic clustering algorithm to detect highly interconnected regions on the basis of large protein-to-protein interacting networks, was conducted to identify the crucial molecular complexes. Eventually, three crucial clusters including cluster 1 (density score = 9.89, nodes = 37, edges = 178), cluster 2 (density score = 6.27, nodes = 31, edges = 91), and cluster 3 (density score = 4.89, nodes = 28, edges = 66) were ascertained (Fig. 6C, D, and F).

The KEGG pathway enrichment demonstrated the significant role of the co-expressed genes within three crucial clusters in the regulation of actin cytoskeleton, endocytosis, Fc gamma R-mediated phagocytosis, Chemokine signaling pathway as well as B cell receptor signaling pathway (Fig. 6B). Meanwhile, the immune-related processes analysis indicated their correlation to Fc receptor-mediated stimulatory pathway, T cell costimulation, antigen processing and presentation of exogenous peptide antigen via MHC class II, and positive regulation of gamma-delta T cell differentiation (Fig. 6E). The discoveries suggested that the genes within crucial molecular clusters played a critical role in the activation and response of immune system, indicating its association with the tumor microenvironment of HCC.

We next intersected 96 co-expressed genes included in three crucial clusters with all immune-related genes (IRGs) to finally obtain sixteen immune-related co-expressed gene signatures (IRCGS) (Fig. 6G). Moreover, the relationship between the expression of IRCGS and infiltration of six classical immune cell subtypes including B cells, CD4^+^ T cells, CD8^+^ T cells, macrophages, neutrophils, and dendritic cells (DC) was evaluated via Pearson's correlation values in the TIMER database. The results indicated a strong correlation between SYK, ZC3HAV1, LYN, HCK, VAV1, PIKC3A, CSK, NCK1, CD86, PPP3CA, TANK, FGFR1, CBL, CYBB, RHOA, and CBLB, and immune cells infiltrating under the adjustment of tumor purity (Table 1).

**Table 1:** The correlation of sixteen immune-related gene markers and immune cell infiltration within various subtypes in the HCC cohort from TCGA database

Genes	Tumor purity			CD8 + T Cell		CD4 + T Cell		Macrophage		Neutrophil		Dendritic cell	
	R	P value		R	P value	R	P value	R	P value	R	P value	R	P value
SYK	− 0.504	****		0.610	****	0.545	****	0.739	****	0.576	****	0.785	****
ZC3HAV1	− 0.18	***		0.247	****	0.335	****	0.366	****	0.451	****	0.426	****
LYN	− 0.358	****		0.251	****	0.378	****	0.415	****	0.573	****	0.437	****
HCK	− 0.518	****		0.620	****	0.449	****	0.700	****	0.619	****	0.810	****
VAV1	− 0.529		****	0.690	****	0.456	****	0.669	****	0.580	****	0.795	****
PIK3CA	0.0358		0.507	0.245	****	0.392	****	0.436	****	0.532	****	0.370	****
CSK	− 0.181		***	0.254	****	0.508	****	0.481	****	0.399	****	0.450	****
NCK1	− 0.059		0.277	0.226	****	0.374	****	0.373	****	0.516	****	0.370	****
CD86	− 0.515		****	0.664	****	0.429	****	0.732	****	0.598	****	0.831	****
PPP3CA	− 0.081		0.132	0.257	****	0.398	****	0.414	****	0.481	****	0.382	****
TANK	0.017		0.758	0.235	****	0.385	****	0.373	****	0.509	****	0.319	****
FGFR1	− 0.399		****	0.300	****	0.449	****	0.485	****	0.391	****	0.408	****
CBL	− 0.152		**	0.328	****	0.573	****	0.526	****	0.559	****	0.518	****
CYBB	− 0.497		****	0.608	****	0.432	****	0.716	****	0.631	****	0.780	****
RHOA	− 0.0201		0.709	0.312	****	0.408	****	0.516	****	0.485	****	0.484	****
CBLB	− 0.096		0.007	0.366	****	0.421	****	0.444	****	0.538	****	0.468	****

R: Pearson’s correlation coefficient. All **P < 0.01, ****P < 0.001, *****P < 0.0001

### Identification of survival-related IRCGS and evaluation of their potential role in the progression of HCC

The K-M curve was performed to assess the predictive value of sixteen IRCGS in OS and DSS probability in the TCGA HCC cohort. And the result showed that HCC patients with higher expression of RHOA and ZC3HAV1 experienced a shorter survival time, however, the patients with higher expression of VAV1 exhibited a superior survival ratio (Additional file 11: Figure S7A-D). Next, the co-expressed correlation of survival-related IRCGS including VAV1, RHOA, and ZC3HAV1, and survival-related gene markers within the STING pathway was further assessed in the HCC cohort from ICGC database (Additional file 11: Figure S7E-G).

**Fig. 7:**
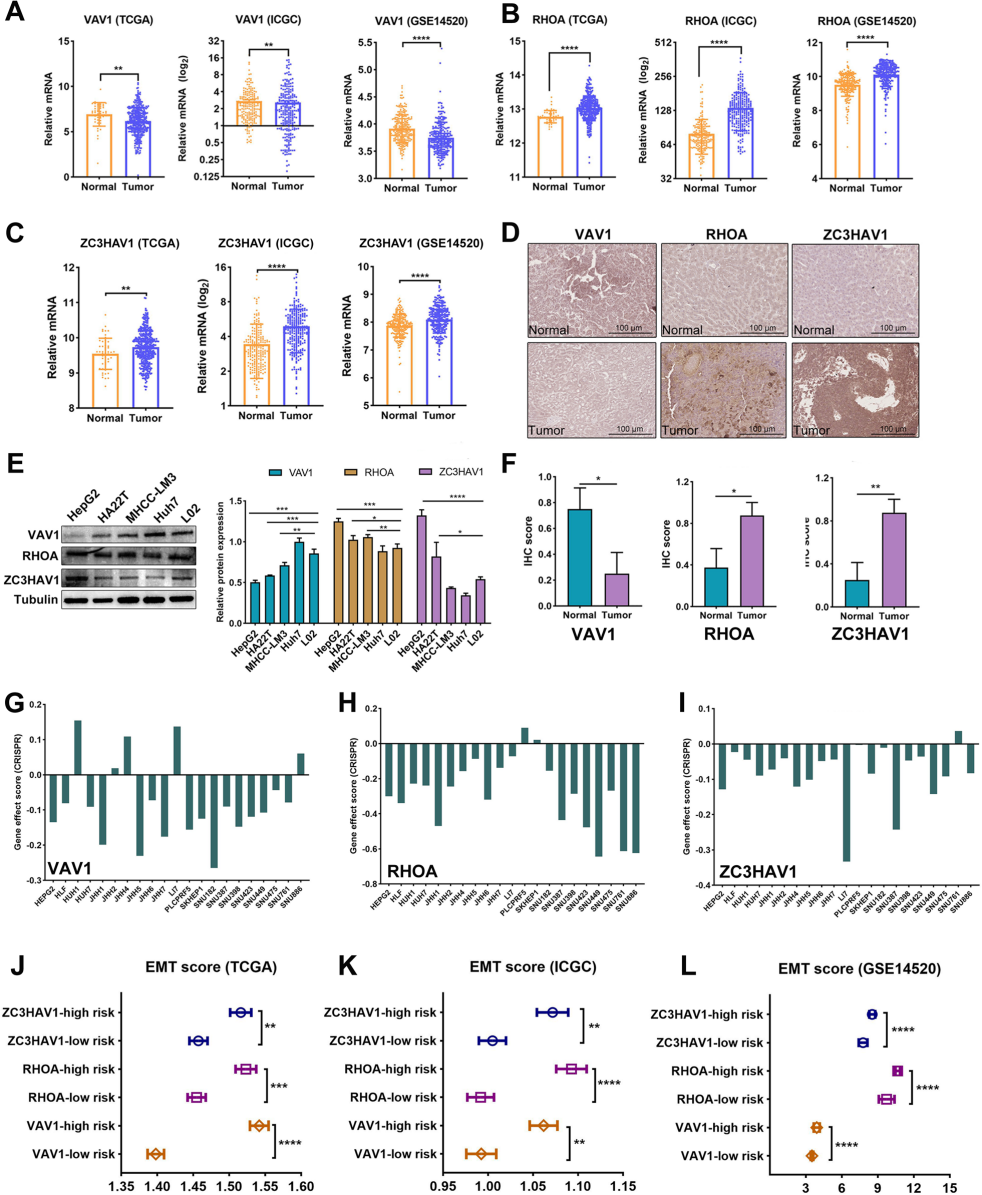
The expression profile of survival-related IRCGS in HCC patients and their significant influence on the progression of HCC. **A–C** The mRNA-expressed level indicated that RHOA and ZC3HAV1 were up-regulated in tumor samples than in normal liver tissues. However, the VAV1 showed the reverse tendency. **D, F** The immunohistochemistry (IHC) was performed to detect the staining intensity and area in tumor samples and adjacent liver tissues from eight clinical patients with HCC. (**D**) indicated the representative staining images of VAV1, RHOA, and ZC3HAV1, and (**F**) was the statistical result. **E** Western blotting was used to determine the protein expression of key IRCGS in human HCC cells and immortalized liver cells. **G–I** The gene depletion assay via CRISPR system showed the gene effect score of key IRCGS in various HCC cell lines from DepMap database. A lower gene effect score indicated a higher possibility that the target gene is essential in a certain cell line, and the 0 score means that the gene is not necessary, correspondingly, -1 is the median value of all pan-essential genes. **J–L** The difference in EMT scores in the low and high expression of VAV1, RHOA, and ZC3HAV1 in HCC datasets from TCGA, ICGC, and GSE14520 were compared, respectively. All *P < 0.05, **P < 0.01, ***P < 0.001, ****P < 0.0001. EMT: epithelial-mesenchymal transition

After that, We investigated the difference in transcriptional expression of VAV1, RHOA, and ZC3HAV1 between tumor samples and adjacent normal liver tissues in various HCC datasets from TCGA, ICGC, and GEO14520. It was indicated that VAV1 expression was higher in normal tissues than in tumor samples, whereas, the elevated expression of RHOA and ZC3HAV1 was observed in the tumor group (Fig. 7A–C). What’s more, the IHC assay was performed to determine the staining intensity of three survival-related IRCGS in eight pairs of real-world tumor samples and adjacent normal liver tissues (Fig. 7D, F, Additional file 12: Figure S8), and western blotting was utilized to detect their difference in protein expression between human HCC and immortalized liver cells (Fig. 7E). The experimental results indicated a consistent trend that has been observed in three public HCC cohorts. Nonetheless, the expression of VAV1, RHOA, and ZC3HAV1 in the tumor group was all up-regulated than that in the normal group in several HCC cohorts from Oncomine database (Table 2).

**Table 2:** The expression difference of VAV1, RHOA, and ZC3HAV1 in transcription level between tumor samples and normal liver tissues from Oncomine database

Genes	Cohorts	Cancer type	Sample counts		Expression type	Fold change	P-value	t-test
			Tumor	Liver				
VAV1	Mas liver	HCC	38	19	Elevated in tumor	1.21	3.58E-06	4.999
RHOA	Chen Liver	HCC	104	76	Elevated in tumor	1.38	1.05E-10	6.743
	Mas Liver	HCC	38	19	Elevated in tumor	1.36	5.78E-05	4.240
	Roessler Liver 2	HCC	225	220	Elevated in tumor	1.57	2.85E-24	10.739
ZC3HAV1	Wurmbach Liver	HCC	35	10	Elevated in tumor	2.18	5.34E-09	7.103
	Chen Liver	HCC	104	76	Elevated in tumor	1.32	5.13E-09	6.011
	Mas Liver	HCC	38	19	Elevated in tumor	1.23	3.47E-09	3.665
	Guichard Liver	HCC	99	86	Elevated in tumor	1.05	9.37E-09	5.034
	Roessler Liver	HCC	22	21	Elevated in tumor	1.25	1.70E-02	2.218
	Roessler Liver 2	HCC	225	220	Elevated in tumor	1.11	6.48E-08	5.371
	Guichard Liver 2	HCC	26	26	Elevated in tumor	1.02	4.00E-02	1.824

Then, the gene effect score of a variety of human HCC cell lines via the CRISPR method from DepMap database indicated a significant role of VAV1, RHOA, and ZC3HAV1 in the process of HCC vulnerability, since the scores from most cell lines were < 0 scores (Fig. 7G–I). More importantly, we divided HCC patients from TCGA, ICGC, and GSE14520 datasets into low- and high-risk subsets based on the best cutoff value of VAV1, RHOA, and ZC3HAV1 expression determined by the K-M method, and found that the EMT scores of survival-related IRCGS in the high-risk group were higher than those within the low-risk group. The findings demonstrated a potential involved mechanism that VAV1, RHOA, and ZC3HAV1 were associated with the prognosis of HCC patients (Fig. 7J–L, Additional file 13: Figure S9).

### Association of survival-related IRCGS and immune cell infiltration within HCC microenvironment

**Fig. 8:**
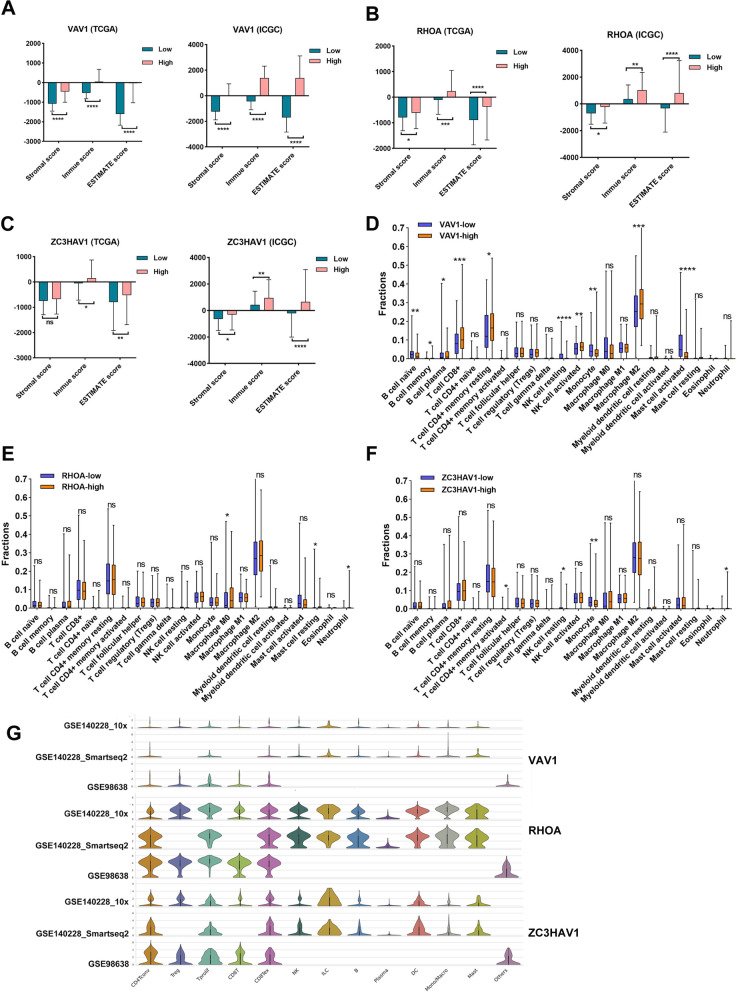
The correlation of survival-related IRCGS and immune cell infiltrates in HCC. **A–C** The stromal, immune, and ESTIMATE scores were calculated across the high and low expression of VAV1, RHOA, and ZC3HAV1 in HCC cohorts from TCGA and ICGC databases. **D–F** The fractional difference in the infiltration of 22 immune cell subtypes between low and high expression of VAV1, RHOA, and ZC3HAV1 from the TCGA HCC cohort was evaluated via CIBERSORT algorithm. **G** The correlation of key IRCGS and the distribution of immune cell subtypes was further determined in three separate single-cell sequencing HCC datasets from TISCH database. TISCH: Tumor Immune Single-cell Hub. All *P < 0.05, **P < 0.01, ***P < 0.001, ****P < 0.0001, ns: no significance

At first, we calculated the stromal, immune, and ESTIMATE scores across HCC datasets from TCGA and ICGC. The results revealed that the high expression of VAV1, RHOA, and ZC3HAV1 was closely related to the HCC microenvironment (Fig. 8A–C). To further understand the relationship between the expressed level of VAV1, RHOA, and ZC3HAV1, and the infiltration of immune cell types, the CIBERSORT algorithm was performed to evaluate the distributed difference of 22 immune cell subtypes in HCC patients within low- and high-risk group stratified by VAV1, RHOA, and ZC3HAV1, respectively. For VAV1 expression, we noticed that B cells, CD8^+^ T cells, CD4^+^ memory resting T cells, activated NK cells, and M2 macrophages exhibited a positive correlation with the expression level. Nevertheless, the naive B cells, resting NK cells, monocyte, and activated mast cells were enriched in the low-risk group (Fig. 8D). Concerning RHOA, the M0 macrophage and neutrophil cells have a higher infiltrating level in HCC patients included in the high-risk group. Conversely, the highly infiltrating resting mast cells were gathered in the low-risk group (Fig. 8E). Similarly, as shown in Fig. 8F, the higher-level enrichment of activated CD4^+^ T cells and neutrophil cells was observed in the population of high-risk group classified by ZC3HAV1. At once, the resting NK cells and monocyte were prone to be aggregated in the patients within the low-risk group. Furthermore, we validated the correlation of VAV1, RHOA, and ZC3HAV1 expression and immune cell infiltration in three single-cell sequencing HCC datasets of GSE140028_10x, GSE140228_Smatseq 2, and GSE98638 from TISCH database (Fig. 8G). The results showed their valuable role in the immune cell infiltration in HCC microenvironment as well (Additional file 14: Figure S10).

### Correlation of survival-related IRCGS to immune checkpoint biomarkers and immunotherapy response in HCC

**Fig. 9:**
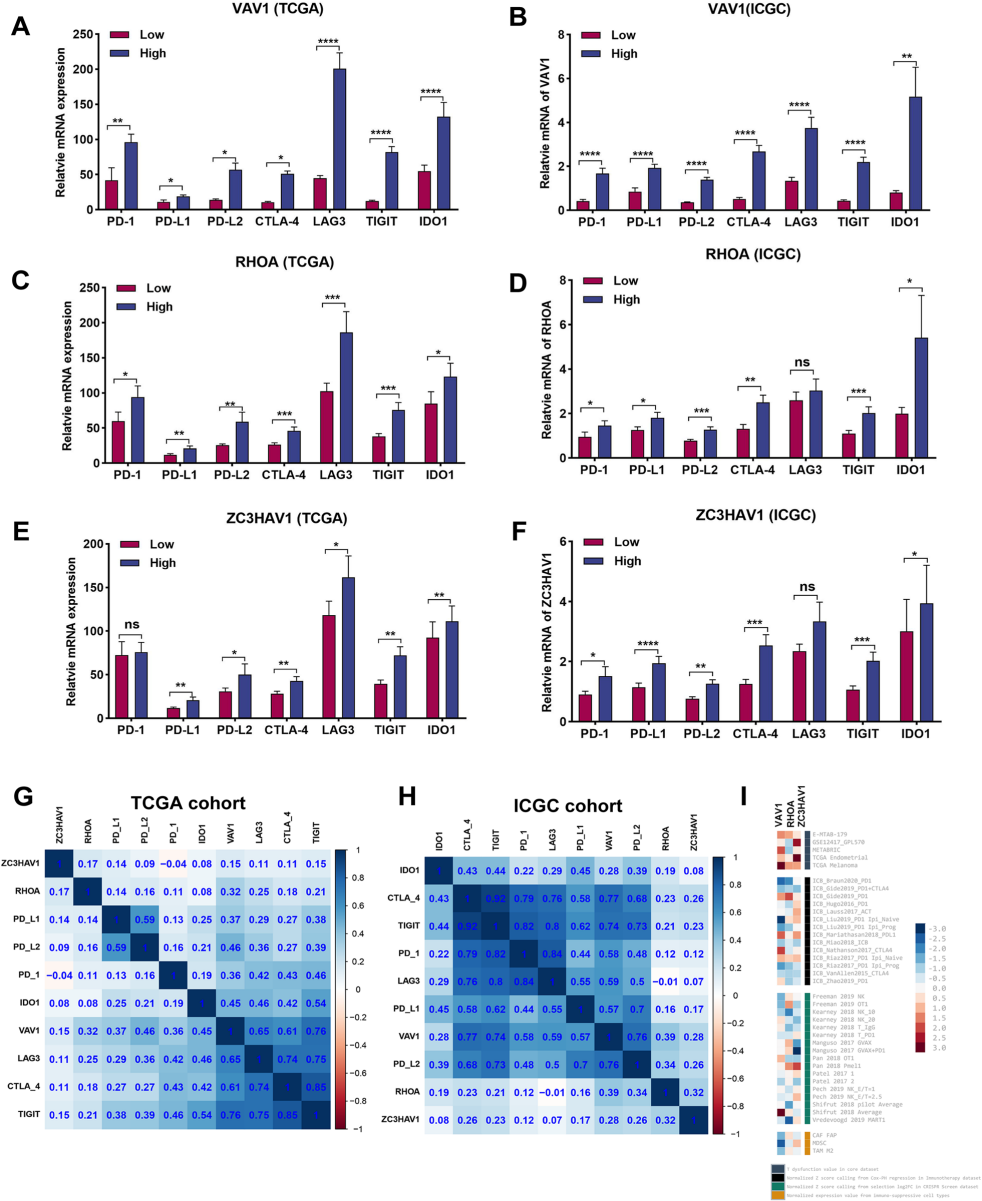
The association between survival-related IRCGS and expression profile of immune checkpoints and immunotherapy efficacy. **A–F** the transcriptional expression profile of most immune checkpoints was significantly increased in the key IRCGS high-expressed group from TCGA and ICGC datasets. **G, H** The co-expressed correlation of key IRCGS and immune checkpoints was presented. **I** The IRCGS including VAV1, RHOA, and ZC3HAV1, was correlated to the immunotherapy response from diverse immunotherapeutic datasets in TIDE database. All *P < 0.05, **P < 0.01, ***P < 0.001, ****P < 0.0001, ns: no significance

To determine the influence of three survival-related IRCGS on the expression of the prominent immune checkpoint molecules of PD-1, PD-L1, PD-L2, CTLA-4, LAG3, TIGIT, and IDO1, we evaluated the expressed difference of immune checkpoint molecules in HCC samples included in the low- and high-risk group stratified by VAV1, RHOA, and ZC3HAV1 from TCGA and ICGC datasets, separately. The results indicated that the expression of all immune checkpoint molecules was up-regulated in the high-risk group of VAV1, RHOA, and ZC3HAV1 (Fig. 9A–F). Next, we explored the co-expressed correlation of immune checkpoint molecules in the two HCC datasets, and the detailed information was presented in Fig. 9G and H.

Furthermore, we used the TIDE algorithm to explore whether the survival-related IRCGS could reflect the immunotherapeutic benefits involved with immune checkpoint inhibitors (ICIs). The result demonstrated a significant value of VAV1, RHOA, and ZC3HAV1 in predicting the therapeutic efficacy of ICIs for patients with various cancer types (Fig. 9I). Additionally, the relationship between the expression of three survival-related IRCGS and the tumor immune dysfunction and exclusion were evaluated in a variety of cancer types, such as melanoma, glioblastoma, and kidney cancer using the tumor cohorts treated with ICIs from TIDE database. It was shown that the expression of VAV1, RHOA, and ZC3HAV1 was associated with the infiltration of CTLs and survival probability of OS and PFS (Table 3).

**Table 3:** The correlation of VAV1, RHOA, and ZC3HAV1 transcriptional expression and immunotherapy response based on various cancer cohorts from TIDE database

Cohort name	Cancer type	Cases	Survival	VAV1			RHOA			ZC3HAV1		
				CTL Cor	Risk	Risk.adj	CTL Cor	Risk	Risk.adj	CTL Cor	Risk	Risk.adj
Braun2020_PD1	Kidney	295	OS	0.074	− 2.675	− 2.358	− 0.056	− 2.039	− 1.904	NA	NA	NA
Gide2019_PD1	Melanoma	41	OS	0.839	− 1.512	1.3	− 0.024	1.642	1.3	0.533	− 1.545	0.302
Gide2019_PD1 + CTLA4	Melanoma	32	OS	0.858	− 1.297	− 1.251	0.085	− 0.833	− 0.919	0.469	− 1.636	− 1.268
Hugo2016_PD1	Melanoma	25	OS	0.835	0.707	− 0.145	− 0.083	− 0.893	− 0.884	0.191	0.692	0.688
Lauss2017_ACT	Melanoma	25	OS	0.872	− 0.439	0.012	− 0.115	− 0.295	− 0.299	0.423	0.711	1.122
Liu2019_PD1	Melanoma	47	OS	0.69	− 2.263	− 1.643	0.114	− 1.411	− 1.184	0.361	− 0.518	0.036
Liu2019_PD1	Melanoma	74	OS	0.59	− 2.201	− 2.874	0.114	− 2.399	− 2.326	0.394	− 1.451	− 1.171
Mariathasan2018_PDL1	Bladder	348	OS	0.708	− 0.249	1.82	0.187	− 0.194	0.198	0.497	0.117	1.306
Miao2018_ICB	Kidney	33	OS	0.599	− 0.928	− 1.108	0.262	0.408	− 0.24	0.298	− 0.89	− 1.023
Nathanson2017_CTLA4	Melanoma	9	OS	0.803	1.784	2.067	0.017	0.602	0.723	0.392	− 0.33	1.435
Nathanson2017_CTLA4	Melanoma	15	OS	0.886	− 2.018	− 0.834	0.291	− 0.134	− 0.534	− 0.195	− 0.067	0.165
Riaz2017_PD1	Melanoma	25	OS	0.833	0.05	1.014	0.298	0.532	0.708	0.453	0.968	0.894
Riaz2017_PD1	Melanoma	26	OS	0.805	− 1.656	− 1.71	0.192	− 1.52	− 1.043	0.293	− 0.712	− 0.637
VanAllen2015_CTLA4	Melanoma	42	OS	0.797	− 2.399	− 0.823	0.16	− 1.757	− 1.446	0.564	− 2.052	− 0.904
Zhao2019_PD1	Glioblastoma	15	OS	0.701	0.546	0.592	− 0.31	− 0.634	− 0.668	0.083	0.904	1.731
Zhao2019_PD1	Glioblastoma	9	OS	0.383	− 1.467	− 1.319	0.409	− 1.749	− 1.511	0.501	− 0.422	− 0.55
Braun2020_PD1	Kidney	295	PFS	0.074	− 0.181	− 0.114	− 0.056	− 0.896	− 0.792	NA	NA	NA
Gide2019_PD1	Melanoma	41	PFS	0.839	− 2.269	1.434	− 0.024	1.9	1.99	0.533	− 1.521	0.883
Gide2019_PD1 + CTLA4	Melanoma	32	PFS	0.858	− 0.774	1.201	0.085	− 0.116	− 0.084	0.469	− 0.334	0.485
Lauss2017_ACT	Melanoma	25	PFS	0.872	− 0.504	0.396	− 0.115	0.842	0.977	0.423	1.353	2.345
Liu2019_PD1	Melanoma	74	PFS	0.59	− 0.891	− 0.512	0.304	0.454	0.525	0.361	0.086	1.035
Liu2019_PD1	Melanoma	47	PFS	0.69	− 2.684	− 1.365	0.304	− 0.245	0.019	0.394	0.742	0.92
Miao2018_ICB	Kidney	33	PFS	0.599	− 1.757	− 2.72	0.262	− 0.472	− 0.474	0.298	− 1.567	− 1.943
Riaz2017_PD1	Melanoma	25	PFS	0.833	0.69	0.821	0.298	0.092	− 0.059	0.453	0.542	0.892
Riaz2017_PD1	Melanoma	26	PFS	0.805	− 2.412	− 1.981	0.192	− 1.363	− 1.222	0.293	− 1.571	− 1.261
VanAllen2015_CTLA4	Melanoma	42	PFS	0.797	− 1.7	− 0.487	0.16	− 1.358	− 0.979	0.564	− 1.041	− 0.012
Zhao2019_PD1	Glioblastoma	15	PFS	0.701	0.028	0.169	− 0.31	− 1.021	− 1.06	0.083	0.507	1.685
Zhao2019_PD1	Glioblastoma	9	PFS	0.383	− 1.939	− 1.63	0.409	− 1.722	− 1.679	0.501	− 1.089	− 1.105

TIDE: tumor immune dysfunction and exclusion. Cor. Pearson’s correlation coefficient. Risk.adj: Risk adjustment. OS: overall survival. PFS: progression-free survival. CTL: cytotoxic T lymphocyte. NA: not applicable

## Discussion

HCC is one of the most frequent malignant tumors and is ranked the second most common cause of cancer-related mortality worldwide. Recently, patients diagnosed with early-stage disease have the chance to be curably treated, with the rapid advancement of surgical strategies, such as intrahepatic surgery, radiofrequency ablation (RFA), transarterial chemoembolization (TACE), and liver transplantation. However, the long-term prognosis of HCC patients is still not satisfactory owing to the high incidence of intra- and extra-hepatic recurrence and metastatic burden [2, 3]. Meanwhile, for patients at the advanced stages, systematic treatments like cell cytotoxic reagents (gemcitabine and oxaliplatin et al.) and molecular-targeted agents (MTAs) like sorafenib have been considered efficient treatments to prevent the pace of HCC progression [5, 27].

STING is a critical signal adaptor that regulated cytosolic DNA-induced innate immune responses via directly recognizing the bacterial cyclic dinucleotides (CDNs) and 3′3′c-GAMP to activate the host immune response. Moreover, the endogenous cGAMP could be produced by cGAS which was a type of cyclic GMP-AMP synthase and a cytosolic DNA sensor. Then, the signaling cascade of cGAS-STING was established, in turn, triggering type I interferons (IFNs), TANK-binding kinase I (TBK1), and interferon regulatory factor 3 (IRF3) et al. [14, 28]. Recently, several studies indicated that the activation of members within the STING pathway was correlated to the immune-active tumor microenvironment (TME), then exhibiting its prognostic value in patients with tumors [14, 18–20].

Generally, cancer cells are more fragile and easily occur chromosomal instability (CIN), which is the primary origin of cytoplasmic DNA in tumor cells and is usually involved with tumor progression, increasing metastatic burden, and resistance to treatment [29]. Nonetheless, the potential impact of the expression and biological function of gene members included in the STING pathway on the initiation, progression, and prognosis of tumors including HCC was still limited known [14, 29]. In the current study, we first downloaded all markers within the STING pathway from MSigDB database including XRCC6, XRCC5, TRIM21, TRECX1, TBK1, STING1, STAT6, PRKDC, NLRP4, NLRC3, MRE11, IRF3, IFI16, DTX4, DDX41, and cGAS. We explored the transcriptional expression profile of each gene in the HCC and adjacent normal liver tissues in various HCC datasets from TCGA, ICGC, and GEO databases. Subsequently, the qPCR assay was operated to detect the expression difference of sixteen members within the STING pathway in HCC cells and immortalized liver cells, which showed a consistent expressed trend with public datasets. After that, we screened out five survival-related markers of NLRC3, STING1, TBK1, TRIM21, and XRCC6 via multivariate Cox regression analysis, and further used them to establish a prognostic nomogram, showing the credible performance in predicting overall survival at 1-, 3-, and 5- years for individual patients. The findings give us knowledge, to a certain degree, to realize the preliminary expression profile of the STING pathway in HCC and its potential predictive role in the prognosis of HCC patients. However, concerning the involved molecular process and regulation mechanisms, in-depth research across in vitro and in vivo models is warranted to uncover in the future.

NLRC3, a member of the NOD-LIKE receptor family, has the features of a central NACHT domain, C-terminal leucine-rich repeat (LRR), and variable interaction domain located in the N-terminal. It was a cytosolic regulator of innate immunity contributing to the activation of innate immune response and functioning as the inhibitor of cellular proliferative and apoptotic capacity [30, 31]. Chen et al. found that NLRC3 depletion was the key step for miR-190b acting as the crucial regulator to promote the growth and metastasis of bladder carcinoma [30]. The stimulator of interferon response cGAMP interactor I (STING1), which is also known as STING or TMEM173, is an endoplasmic reticulum (ER) protein. Recent evidence indicated that STING1 played a crucial role in enhancing innate immune signal transduction induced by host DNA damage and pathogen infection. Moreover, STING1 participated in several cancer-related processes such as apoptosis, pyroptosis, necroptosis, and ferroptosis, implying its potential role in molecular targeting for preventing the progression of malignancies [32, 33].

At present, it was reported that the tripartite motif-containing protein 21 (TRIM21) was a downstream biomarker within the STING pathway and was included in the tripartite motif-containing (TRIM) superfamily, with the evolutionally conserved domain structure of N-terminal RING area related to E3 ubiquitin ligase activity [34]. Then, it could take part in the process of ubiquitylation and proteasome-dependent degradation mediated by ubiquitin ligases, resulting in its crucial impact in controlling cellular protein expression and clearance. Zhow et al. found that the reduced expression of TRIM21 was involved with an unfavorable outcome for patients with breast cancer [35]. Similarly, Wang et al. indicated that TRIM21 accelerated the progression of HCC by inhibiting the anti-antioxidant signaling of p62-Keap1-Nrf2 in vivo mice model [34]. In parallel, the STING pathway also activates XRCC6 to mediate the cellular process of DNA repair which played a crucial role in maintaining cell viability and genome stability. Some evidence demonstrated the correlation between dysfunction of XRCC6 and tumorigenesis of various cancer types, such as breast cancer and colorectal cancer [36, 37].

With regard to the TANK-binding kinase 1(TBK1), it was a serine/threonine kinase involved in the induction of IFN-I produce, which was activated by STING1 with being regulated by the second messenger of cGAMP [38, 39]. Recent studies suggested that TBK1 exhibited a significant role in mediating the process of survival probability and development in Kras-independent cancer cells [40–42]. In this study, the findings revealed that gene markers of NLRC3, STING1, TBK1, TRIM21, and XRCC6 are independent indicators to predict inferior survival probability for individual HCC patients. Furthermore, the wound-healing assay and transwell migratory system suggested that the up-regulation of TBK1 promoted the migratory ability of HCC cells, which may be a potential involved molecular mechanism. Whereas numerous more thoughtful and stringent investigations of in vivo and in vitro approaches were required to fully elaborate their specific functions and the involved regulation mechanisms in the progression and prognosis of HCC.

Nowadays, it was widely accepted that the liver is a classic tolerogenic organ showing a specific microenvironment to prevent the overaction of the immune system and antigens originating from food and bacterial products [5, 9]. At one time, HCC cells could evade host immune surveillance via several mechanisms including silencing the expression of tumor-related antigens, increasing the infiltration of suppressive immune cells, such as MDSC and tumor-associated macrophages (TAM), and expressing immunoinhibitory factors like PD-1, PD-L1, CTLA-4, and indoleamine 2, 3-dioxygenase (IDO1) [43–45]. Even under these immunosuppressive conditions, during the past decade, immunotherapies have obtained unprecedented efficacy in anti-tumor treatments including melanoma, breast cancer, and HCC et al. However, the majority of patients accounting for more than 70% still didn’t benefit from immune-based therapies [11, 44]. Therefore, more studies focused on the tumor microenvironment (TME) of HCC were imperative to better understand its tumorigenesis and uncover potential novel targets for future immunotherapeutic treatments.

In the current study, we identified three survival-related IRCGS, namely VAV1, RHOA, and ZC3HAV1, and indicated that higher expression of VAV1 was preferable to occur in normal liver tissues. Reversely, the up-regulated expression of RHOA and ZC3HAV1was detected in the HCC samples. Subsequently, the gene effect score underlying CRISPR method and EMT scores demonstrated the crucial function of VAV1, RHOA, and ZC3HAV1 in the tumorigenesis and progression of HCC from diverse aspects. Previous studies reported that VAV1 is one member of a novel family of DbI guanine nucleotide exchange factors of the GTPases, extremely dependent on tyrosine phosphorylation to execute its function. Several recent studies revealed its elevated expression in several human malignant tumors, like pancreatic cancer, suggesting its association with the carcinogenesis of human cancers [46, 47]. Meanwhiles, it could inhibit E-cadherin expression through transactivation of Snail and Slug markers, uncovering its potential function in the process of EMT [47]. RHOA was one isoform of Rho GTPases that belong to the small GTPase family of proteins (~ 21 kDa). These proteins have been found to involve in several significant cancer-related processes in mammalian cells, such as growth, migration, and EMT. However, whether its aberrant expression is implicated in HCC progress still remains unclear [48, 49].

At present, it was clear that ZC3HAV1 is a type of zinc-finger antiviral protein, which goes by the name of ARTD13 or PARP13, classified into the PARP protein family. The current evidence reported that PARP protein families were involved in the development of various diseases like pancreatic cancer [50]. Whereas, the biological function of ZC3HAV1 and its potential regulatory mechanisms in HCC are not clear yet. Furthermore, the investigation focused on the relationship between three survival-related IRCGS and tumor microenvironment including HCC is still limited. Here, the findings showed that the patients with increased expression of VAV1, RHOA, and ZC3HAV1, exhibited higher immune-related scores using ESTIMATE algorithms. More importantly, the elevated expression of key IRCGS indicated the correlation to immune cell infiltration including CD8^+^ T cells, M2 macrophages, and neutrophil cells, up-regulated expression of immune checkpoint molecules, and benefit patients from immunotherapeutic treatments in various cancer types. However, the involved molecular processes and regulation mechanisms that VAV1, RHOA, and ZC3HAV1 participated in the progression of HCC via meddling in the composition and functional status of the tumor microenvironment, are not sufficient experimentally explored, leading to the limitation of this study. It is required more scientific evidence in future research to elucidate.

## Conclusion

In all, we comprehensively analyzed the expression and the prognostic value of the STING pathway in HCC cohorts from TCGA, ICGC, and GEO datasets. Five survival-related biomarkers of STING signaling were determined and further established a prognostic nomogram to predict survival probability for individual HCC patients, showing a credible performance. Moreover, three survival-related IRCGS were screened out and demonstrated a significant role in the infiltration of various immune cell subtypes and response to immunotherapy. All these findings enriched our knowledge to understand the tumorigenesis and progression of HCC and give potential molecular targets for immunotherapeutic treatments in the future.

## Supplementary Information

**Additional file 1: Table S1.** The clinical features of patients with hepatocellular carcinoma (HCC) from TCGA, ICGC, and GEO databases.

**Additional file 2: Table S2.** The primers of qPCR assay to detect the transcriptional expression of gene markers within STING pathway in HCC.

**Additional file 3: Table S3.** Multivariate Cox regression analysis to determine the prognostic factors associated with OS within STING pathway in HCC cohort from various databases.

**Additional file 4: Figure S1.** The calibration curves at 1-year, 3-year, and 5-year time points were drawn in TCGA-all (A-C) and ICGC (D, E) HCC datasets. The 5-year follow-up data for the ICGC dataset currently is deficient.

**Additional file 5: Figure S2.** The co-expressed correlation of gene signature of NRLC3, STING1, TBK1, TRIM21, and XRCC6 in HCC patients from TCGA-training (A), TCGA-all (B), and ICGC (C), respectively.

**Additional file 6: Figure S3.** X-title software was utilized to determine the cut-off value of risk score to stratify HCC patients from TCGA-training cohort into low- and high-risk groups. The risk score calculated by gene signature of NRLC3, STING1, TBK1, TRIM21, and XRCC6, was associated with survival probability.

**Additional file 7: Figure S4.** The correlation of risk score calculated by gene signature of NRLC3, STING1, TBK1, TRIM21, and XRCC6, and survival probability and disease progression respectively in patients from TCGA-all (A-C) and ICGC cohort (D-F). All *: P < 0.05, **: P < 0.01, ***: P < 0.001.

**Additional file 8: Figure S5.** The difference of transcriptional expression for NLRC3 and TBK1 between HCC samples and adjacent normal liver tissues in GSE84005 dataset. ***: P < 0.001.

**Additional file 9: Figure S6.** The top 20 co-expressed gene signatures of each survival-related markers of the STING pathway (A-E) and their functional enrichment analysis including KEGG pathways (F) and immune processes (G). R: Pearson’s correlation coefficient. KEGG: Kyoto Encyclopedia of Genes and Genomes.

**Additional file 10: Table S4.** Pearson's correlation coefficient of overlapped co-expressed genes of NLRC3, STING1, TRIM21, TBK1, and XRCC6 from UALCAN database.

**Additional file 11: Figure S7.** Integrative analysis of the prognostic value of IRCGS in HCC patients and validation of its co-expressed correlation with five survival-related members of the STING pathway. K-M curve was performed to screen out three key IRCGS significantly associated with OS and DSS in the HCC cohort from TCGA database (A-D). The HCC dataset from ICGC database was used to validate the reliability of the co-expressed correlation of key IRCGS, namely VAV1 (E), RHOA (F), and ZC3HAV1 (G), and survival-related members of STING signal path, namely NLRC3, STING1, TBK1, TRIM21, and XRCC6. K-M curve: Kaplan–Meier curve. IRCGS: immune-related co-expressed gene signatures.

**Additional file 12: Figure S8.** The composition of immunohistochemistry (IHC) staining intensity of VAV1 (A), RHOA (B), and ZC3HAV1(C) in eight clinical tumor samples from patients with HCC and their adjacent liver tissues.

**Additional file 13: Figure S9.** The transcriptional expression of VAV1, RHOA, and ZC3HAV1 was positively correlated to the distribution of EMT scores in the HCC cohort from TCGA (A, D, and G) and ICGC (B, E, and H) database, and GSE14520 (C, F, and I) dataset. EMT: epithelial-mesenchymal transition. r: Pearson's correlation coefficient.

**Additional file 14: Figure S10.** The relationship between transcriptional expression of VAV1 (A), RHOA (B), and ZC3HAV1 (C) and the infiltration of immune cell subtypes in three independent single-cell sequencing HCC datasets from TISCH database. TISCH: Tumor Immune Single-cell Hub.

**Additional file 15: Figure S11.** The raw results of western blotting for the indicated gene.

## Data Availability

The original contributions presented in the current research have been already included in the article or supplementary materials. Further inquiries could directly contact the corresponding author.

## Abbreviations

HCC: Hepatocellular carcinoma
Anti-PD-1: Anti-programmed cell death protein 1
HBV: Hepatitis B virus
HCV: Hepatitis C virus
IRCGS: Immune-related co-expressed gene signatures
TACE: Transarterial chemoembolization
OS: Overall survival
PFS: Progression-free survival
PRRs: Pattern recognition receptors
cGAS: Cyclic GMP-AMP synthase
NSCLC: Non-small cell lung cancer
EMT: Epithelial-mesenchymal transformation
ROC: Time-dependent receiver operating characteristic
AUC: Area under the curve
CRISPR: Clustered regularly interspaced short palindromic repeats
DC: Dendritic cells
ATCC: American Type Culture Collection
CCTCC: The China Center for Type Culture Collection
SDS-PAGF: Sodium dodecyl sulphate–polyacrylamide gel electrophoresis
HRP: Horseradish peroxidase
DSS: Disease-specific survival
DFS: Disease-free survival
AJCC: The American Joint Committee on Cancer
DC: Dendritic cells
ICIs: Immune checkpoint inhibitors
RFA: Radiofrequency ablation
MTAs: Molecular-targeted agents
IDO1: Indoleamine 2, 3-dioxygenase
CIN: Chromosomal instability
ER: Endoplasmic reticulum
