# Supplementary material for: STING pathway contributes to the prognosis of hepatocellular carcinoma and identification of prognostic gene signatures correlated to tumor microenvironment
Source: Cancer Cell Int. 2022 Oct 12;22:314. doi: 10.1186/s12935-022-02734-4 (PMC9554977; doi:10.1186/s12935-022-02734-4)
Supplement: Supplementary file 1 — Additional file 1: Table S1. The clinical features of patients with hepatocellular carcinoma (HCC) from TCGA, ICGC, and GEO databases. [file 12935_2022_2734_MOESM1_ESM.docx]

**Table S1** The clinical features of patients with hepatocellular carcinoma (HCC) from TCGA, ICGC, and GEO databases.

| **Clinical features** | **Count (%)** | | | |
| --- | --- | --- | --- | --- |
|  | **TCGA_training (n=183)** | **TCGA (n=366)** | **ICGC (n=243)** | **GSE14520 (n=242)** |
| **status** |  |  |  |  |
| alive | 119 (65.0) | 237 (64.8) | 182 (74.9) | 146 (60.3) |
| dead | 64 (35.0) | 129 (35.2) | 61 (25.1) | 96 (39.7) |
| **age^a^** |  |  |  |  |
| ＜ median | 91 (49.7) | 175 (47.8) | 120 (49.40 | 108 (44.6) |
| ≥median | 92 (50.3) | 190 (51.9) | 123 （50.6） | 134 (55.40 |
| missing |  | 1 (0.3) |  |  |
| **sex** |  |  |  |  |
| male | 114 (62.3) | 246 (67.2) | 182 (74.9) | 211 (87.2) |
| female | 69 (37.7) | 120 (32.8) | 61 (25.1) | 31 (12.8) |
| **TNM stage** |  |  |  |  |
| I | 86 (47.0) | 169 (46.2) | 36 (14.8) | 96 (39.7) |
| II | 41 (22.4) | 86 (23.5) | 110 (45.3) | 78 (32.2) |
| III | 42 (23.0) | 84 (23.0) | 76 (31.3） | 51 (21.1) |
| IV | 3 (1.6) | 6 (1.6) | 21 (8.60 | 0 (0.0) |
| missing | 11 (6.0) | 21 (5.7) |  | 17 (7.0) |
| **T stage** |  |  |  |  |
| 1 | 92 (50.3) | 178 (48.60 |  |  |
| 2 | 46 (25.1) | 93 (25.4) |  |  |
| 3 | 38 (20.8) | 79 (21.6) |  |  |
| 4 | 6 (3.3) | 13 (3.6) |  |  |
| missing | 1 (0.5) | 3 (0.8) |  |  |
| **N stage** |  |  |  |  |
| N0 | 124 (67.8) | 254 (69.4) |  |  |
| N1 | 0 (0.0) | 0 (0.0) |  |  |
| missing | 59 (32.2) | 112 (30.6) |  |  |
| **M stage** |  |  |  |  |
| M0 | 128 (69.9) | 263 (71.9) |  |  |
| M1 | 1 (0.5) | 4 (1.1) |  |  |
| missing | 54 (29.5) | 99 (27.0) |  |  |
| **tumor size** |  |  |  |  |
| ≤ 5cm |  |  |  | 153 (63.20 |
| ＞5cm |  |  |  | 88 (36.4) |
| missing |  |  |  | 1 (0.4) |
| **multinodular** |  |  |  |  |
| yes |  |  |  | 52 (21.5) |
| no |  |  |  | 190 (78.5) |

a: the median age for TCGA, ICGC and GSE14520 cohort was 61, 69, and 50 years old, separately.
