# Supplementary material for: STING pathway contributes to the prognosis of hepatocellular carcinoma and identification of prognostic gene signatures correlated to tumor microenvironment
Source: Cancer Cell Int. 2022 Oct 12;22:314. doi: 10.1186/s12935-022-02734-4 (PMC9554977; doi:10.1186/s12935-022-02734-4)
Supplement: Supplementary file 2 — Additional file 2: Table S2. The primers of qPCR assay to detect the transcriptional expression of gene markers within STING pathway in HCC. [file 12935_2022_2734_MOESM2_ESM.docx]

**Table S2** The primers of qPCR assay to detect the transcriptional expression of gene markers within STING pathway in HCC.

| **Description** | **Gene ID** | **Species** | **Primer sequencing (5'-3')** | |
| --- | --- | --- | --- | --- |
|  |  |  | **Forward** | **Reverse** |
| **XRCC6** | 2547 | Human | GGTTTCAAGCCGTTGGTACTGC | CTCCAGACACTTGATGAGCAGAG |
| **XRCC5** | 7520 | Human | GTTCTAAAGGTCTTTGCAGCAAGA | AAAAGCCACGCCGACTTGAGGA |
| **TRIM21** | 6737 | Human | CAGAACTCAGGAGTGTGTGCCA | TCCAAGCCTCACTTGTCTCCGA |
| **TREX1** | 11277 | Human | GCATCTGTCAGTGGAGACCACA | CAGTGGTTGTGACAGCAGATGG |
| **TBK1** | 29110 | Human | CAACCTGGAAGCGGCAGAGTTA | ACCTGGAGATAATCTGCTGTCGA |
| **STING1** | 340061 | Human | CCTGAGTCTCAGAACAACTGCC | GGTCTTCAAGCTGCCCACAGTA |
| **STAT6** | 6778 | Human | CCTTGGAGAACAGCATTCCTGG | GCACTTCTCCTCTGTGACAGAC |
| **PRKDC** | 5591 | Human | GCGCCATATCTGTCATCTGCTG | TTATAGCGGCGCTTCAGGTCGA |
| **NLRP4** | 147945 | Human | GACTTGGTGGTTTCTGCCTACTG | GTGATGCCAACAGATGAGGCTG |
| **NLRC3** | 197358 | Human | TGGCAGTGAGAGAAAACCGCAC | CTAAGCTGGTGAGGCTCCTGTT |
| **MRE11** | 4361 | Human | CAGCAACCAACAAAGGAAGAGGC | GAGTTCCTGCTACGGGTAGAAG |
| **IRF3** | 3661 | Human | TCTGCCCTCAACCGCAAAGAAG | TACTGCCTCCACCATTGGTGTC |
| **DTX4** | 2320 | Human | GTCCGTGAAAGAGCAGATGGAG | GTAGCGACAGATGTCTAGGCTG |
| **CGAS** | 115004 | Human | AGGAAGCAACTACGACTAAAGCC | CGATGTGAGAGAAGGATAGCCG |
| **DDX41** | 51428 | Human | GTCCGTGAAAGAGCAGATGGAG | GTAGCGACAGATGTCTAGGCTG |
| **IFI16** | 3428 | Human | GATGCCTCCATCAACACCAAGC | CTGTTGCGTTCAGCACCATCAC |

qPCR: quantitative real-time polymerase chain reaction.
