# Supplementary material for: STING pathway contributes to the prognosis of hepatocellular carcinoma and identification of prognostic gene signatures correlated to tumor microenvironment
Source: Cancer Cell Int. 2022 Oct 12;22:314. doi: 10.1186/s12935-022-02734-4 (PMC9554977; doi:10.1186/s12935-022-02734-4)
Supplement: Supplementary file 3 — Additional file 3: Table S3. Multivariate Cox regression analysis to determine the prognostic factors associated with OS within STING pathway in HCC cohort from various databases. [file 12935_2022_2734_MOESM3_ESM.docx]

**Table S3** Multivariate Cox regression analysis to determine the prognostic factors associated with OS within STING pathway in HCC cohort from various databases.

| **Gene markers** | **transcriptional expression** | **HCC cohort (TCGA)** | | |  | **HCC cohort (ICGC)** | | |  | **HCC cohort (GSE14520)** | | |
| --- | --- | --- | --- | --- | --- | --- | --- | --- | --- | --- | --- | --- |
|  |  | **HR** | **95% CI** | ***P* value** |  | **HR** | **95% CI** | ***P* value** |  | **HR** | **95% CI** | ***P* value** |
| XRCC6 | low | reference |  |  |  | reference |  |  |  | reference |  |  |
|  | high | 1.17 | 0.72-1.90 | 0.522 |  | 1.01 | 1.00-1.02 | 0.015 |  | 1.53 | 0.93-2.54 | 0.097 |
| XRCC5 | low | reference |  |  |  | reference |  |  |  | reference |  |  |
|  | high | 1.54 | 0.95-2.50 | 0.082 |  | 1.00 | 0.98-1.02 | 0.76 |  | 1.12 | 0.73-1.73 | 0.604 |
| TRIM21 | low | reference |  |  |  | reference |  |  |  | reference |  |  |
|  | high | 1.34 | 0.97-1.86 | 0.076 |  | 0.88 | 0.79-0.98 | 0.023 |  | 0.55 | 0.35-0.88 | 0.012 |
| TBK1 | low | reference |  |  |  | reference |  |  |  | reference |  |  |
|  | high | 1.17 | 0.69-2.01 | 0.557 |  | 1.23 | 1.07-1.41 | 0.003 |  | 0.86 | 0.57-1.30 | 0.473 |
| STAT6 | low | reference |  |  |  | reference |  |  |  | reference |  |  |
|  | high | 0.79 | 0.62-1.02 | 0.068 |  | 1.01 | 0.99-1.02 | 0.505 |  | 0.92 | 0.67-1.25 | 0.579 |
| PRKDC | low | reference |  |  |  | reference |  |  |  | reference |  |  |
|  | high | 1.26 | 0.95-1.67 | 0.102 |  | 1.01 | 0.95-1.07 | 0.716 |  | 1.1 | 0.82-1.47 | 0.524 |
| MRE11 | low | reference |  |  |  | reference |  |  |  | reference |  |  |
|  | high | 1.03 | 0.76-1.40 | 0.843 |  | 0.78 | 0.59-1.03 | 0.075 |  | 1.2 | 0.83-1.74 | 0.327 |
| IRF3 | low | reference |  |  |  | reference |  |  |  | reference |  |  |
|  | high | 1.13 | 0.82-1.57 | 0.456 |  | 1.00 | 0.98-1.02 | 0.865 |  | 1.17 | 0.78-1.76 | 0.442 |
| IFI16 | low | reference |  |  |  | reference |  |  |  | reference |  |  |
|  | high | 1.01 | 0.80-1.26 | 0.951 |  | 1.01 | 0.98-1.04 | 0.716 |  | 1.01 | 0.82-1.25 | 0.915 |
| DTX4 | low | reference |  |  |  | reference |  |  |  | reference |  |  |
|  | high | 1.01 | 0.89-1.14 | 0.933 |  | 1.02 | 0.98-1.06 | 0.368 |  | 1.00 | 0.80-1.26 | 0.985 |
| DDX41 | low | reference |  |  |  | reference |  |  |  | reference |  |  |
|  | high | 1.11 | 0.72-1.72 | 0.635 |  | 1.02 | 0.98-1.06 | 0.327 |  | 0.84 | 0.59-1.19 | 0.327 |
| CGAS | low | reference |  |  |  | reference |  |  |  | reference |  |  |
|  | high | 1.09 | 0.92-1.28 | 0.328 |  | 1.02 | 0.84-1.24 | 0.84 |  |  |  |  |
| NLRP4 | low | reference |  |  |  | reference |  |  |  | reference |  |  |
|  | high | 1.06 | 0.92-1.21 | 0.415 |  | 0.98 | 0.93-1.04 | 0.554 |  |  |  |  |
| NLRC3 | low | reference |  |  |  | reference |  |  |  | reference |  |  |
|  | high | 0.58 | 0.45-0.76 | <0.001 |  | 0.44 | 0.20-0.98 | 0.045 |  |  |  |  |
| STING1 | low | reference |  |  |  | reference |  |  |  | reference |  |  |
|  | high | 1.12 | 0.84-1.49 | 0.441 |  | 1.05 | 1.00-1.10 | 0.033 |  |  |  |  |
| TREX1 | low | reference |  |  |  | reference |  |  |  | reference |  |  |
|  | high | 0.97 | 0.82-1.16 | 0.758 |  | 0.94 | 0.84-1.05 | 0.28 |  |  |  |  |

OS: overall survival. HR: harzard ratio. CI: confidence interval.
