# Supplementary figures and images for: STING pathway contributes to the prognosis of hepatocellular carcinoma and identification of prognostic gene signatures correlated to tumor microenvironment
Source: Cancer Cell Int. 2022 Oct 12;22:314. doi: 10.1186/s12935-022-02734-4 (PMC9554977; doi:10.1186/s12935-022-02734-4)

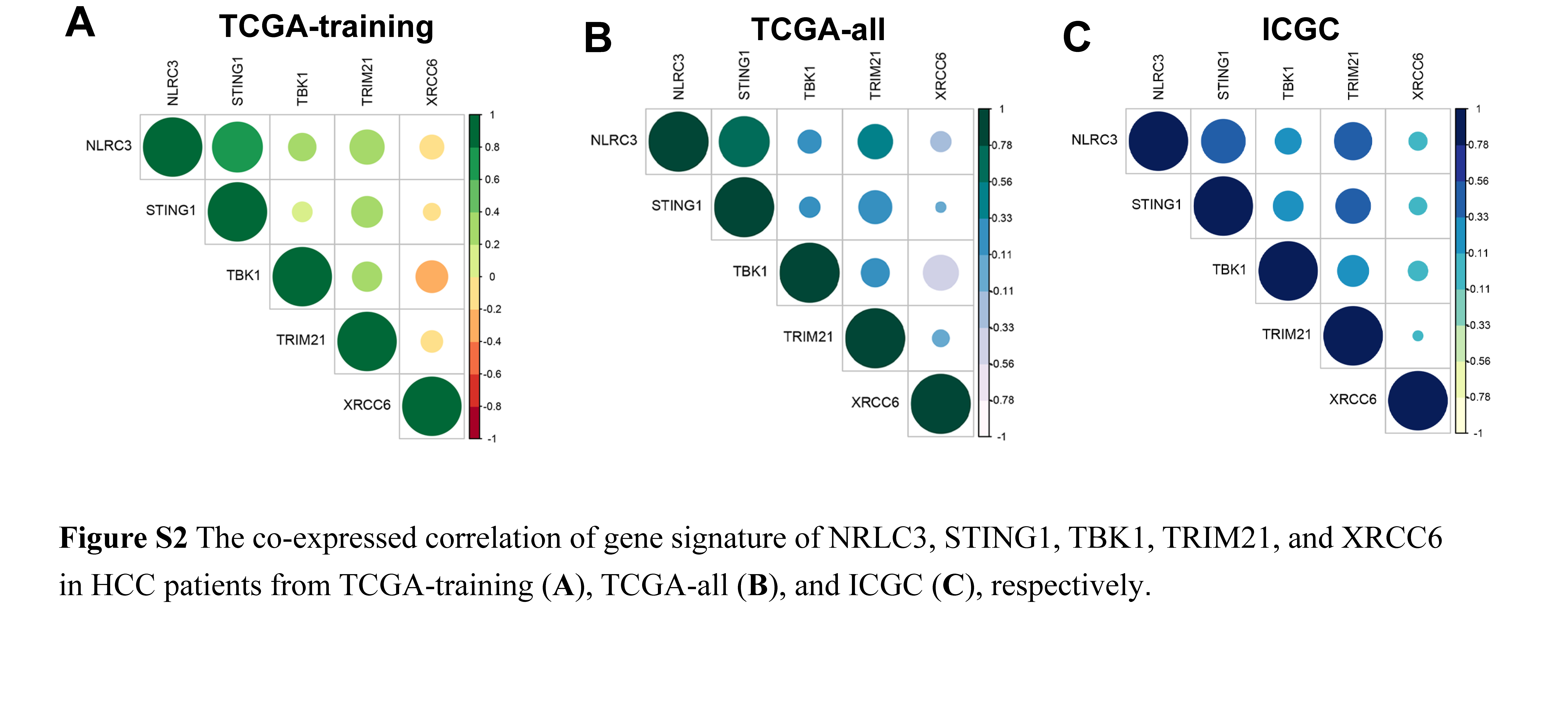

Supplement: Supplementary file 5 — Additional file 5: Figure S2. The co-expressed correlation of gene signature of NRLC3, STING1, TBK1, TRIM21, and XRCC6 in HCC patients from TCGA-training (A), TCGA-all (B), and ICGC (C), respectively. [file 12935_2022_2734_MOESM5_ESM.tif]

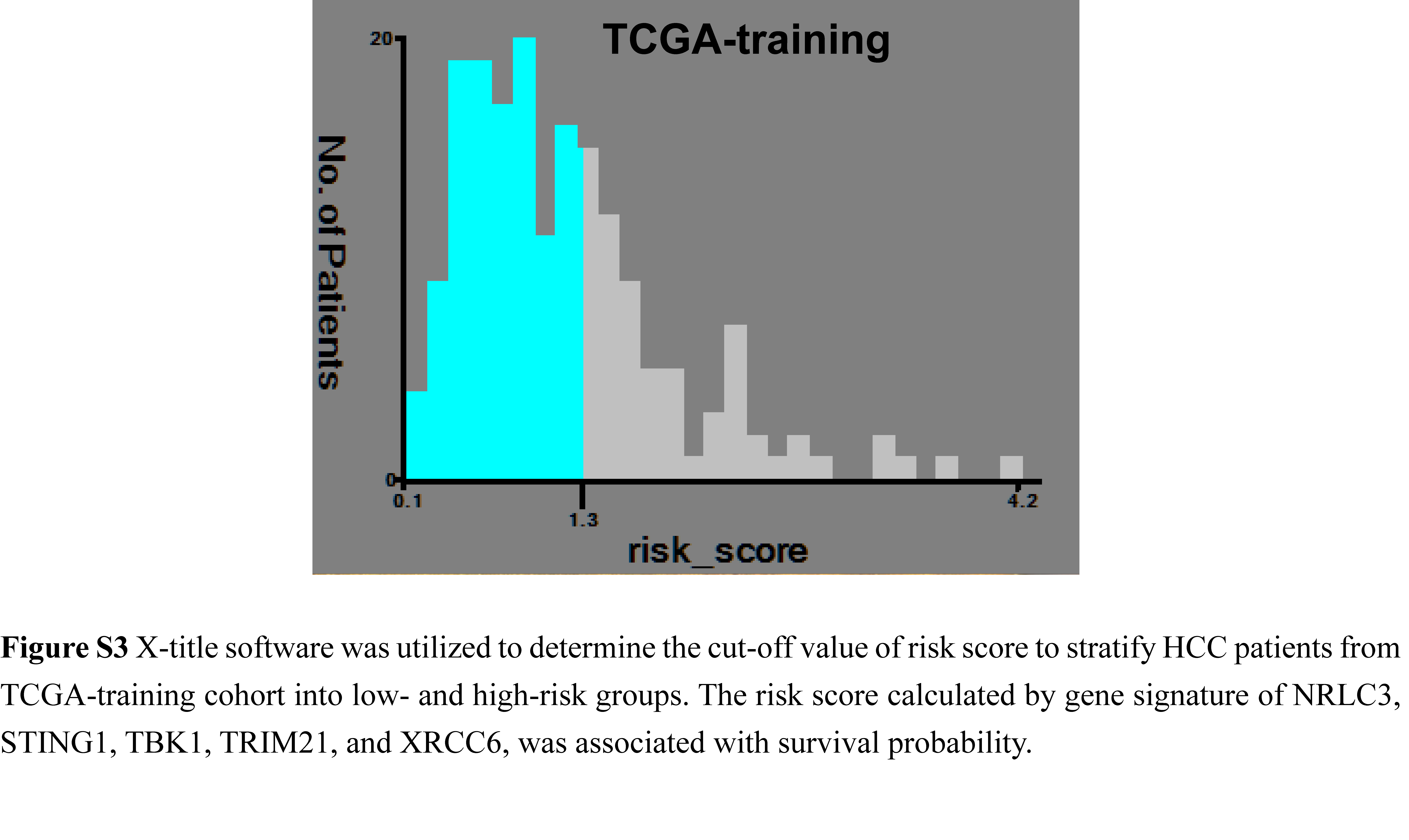

Supplement: Supplementary file 6 — Additional file 6: Figure S3. X-title software was utilized to determine the cut-off value of risk score to stratify HCC patients from TCGA-training cohort into low- and high-risk groups. The risk score calculated by gene signature of NRLC3, STING1, TBK1, TRIM21, and XRCC6, was associated with survival probability. [file 12935_2022_2734_MOESM6_ESM.tif]

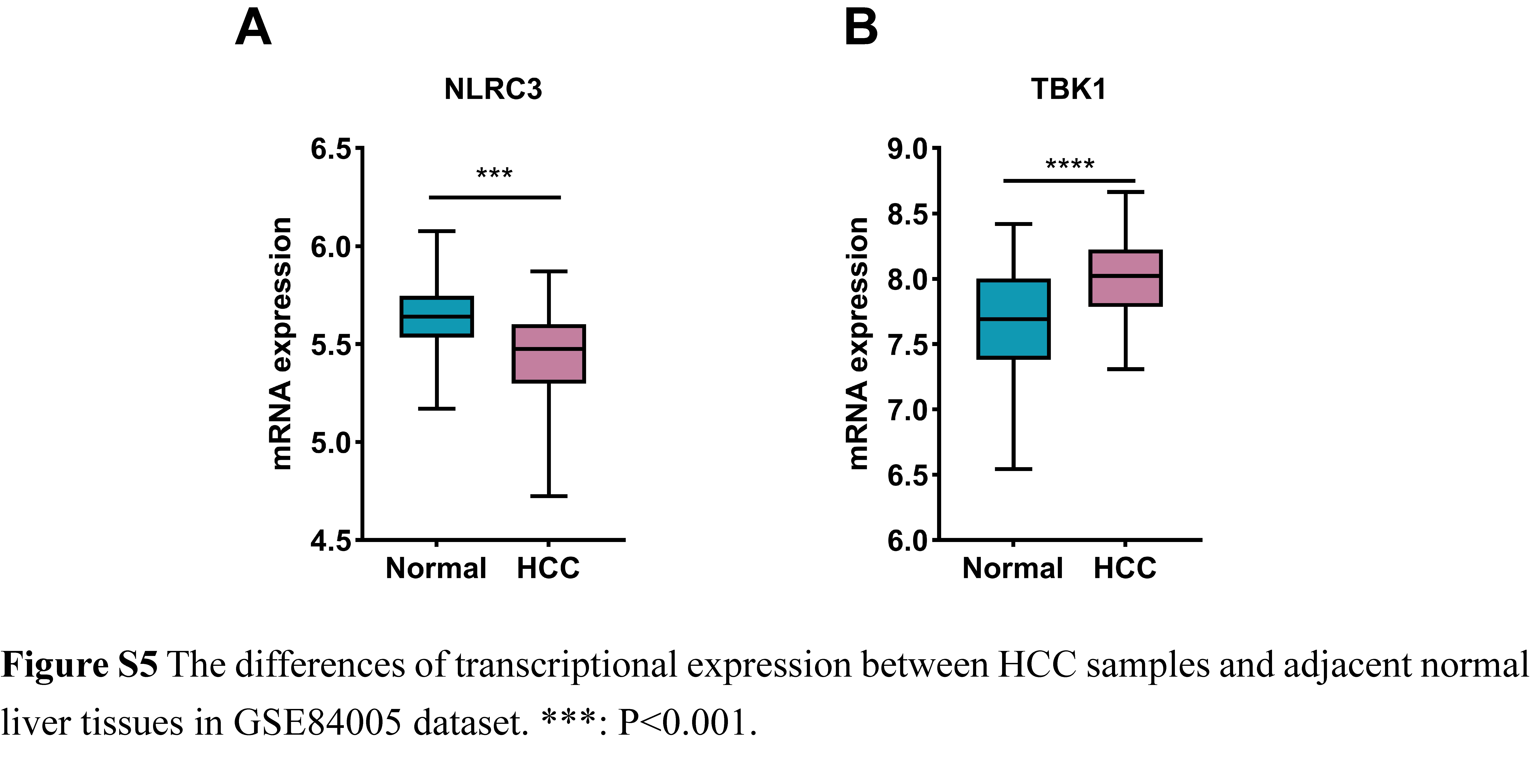

Supplement: Supplementary file 8 — Additional file 8: Figure S5. The difference of transcriptional expression for NLRC3 and TBK1 between HCC samples and adjacent normal liver tissues in GSE84005 dataset. ***: P < 0.001. [file 12935_2022_2734_MOESM8_ESM.tif]

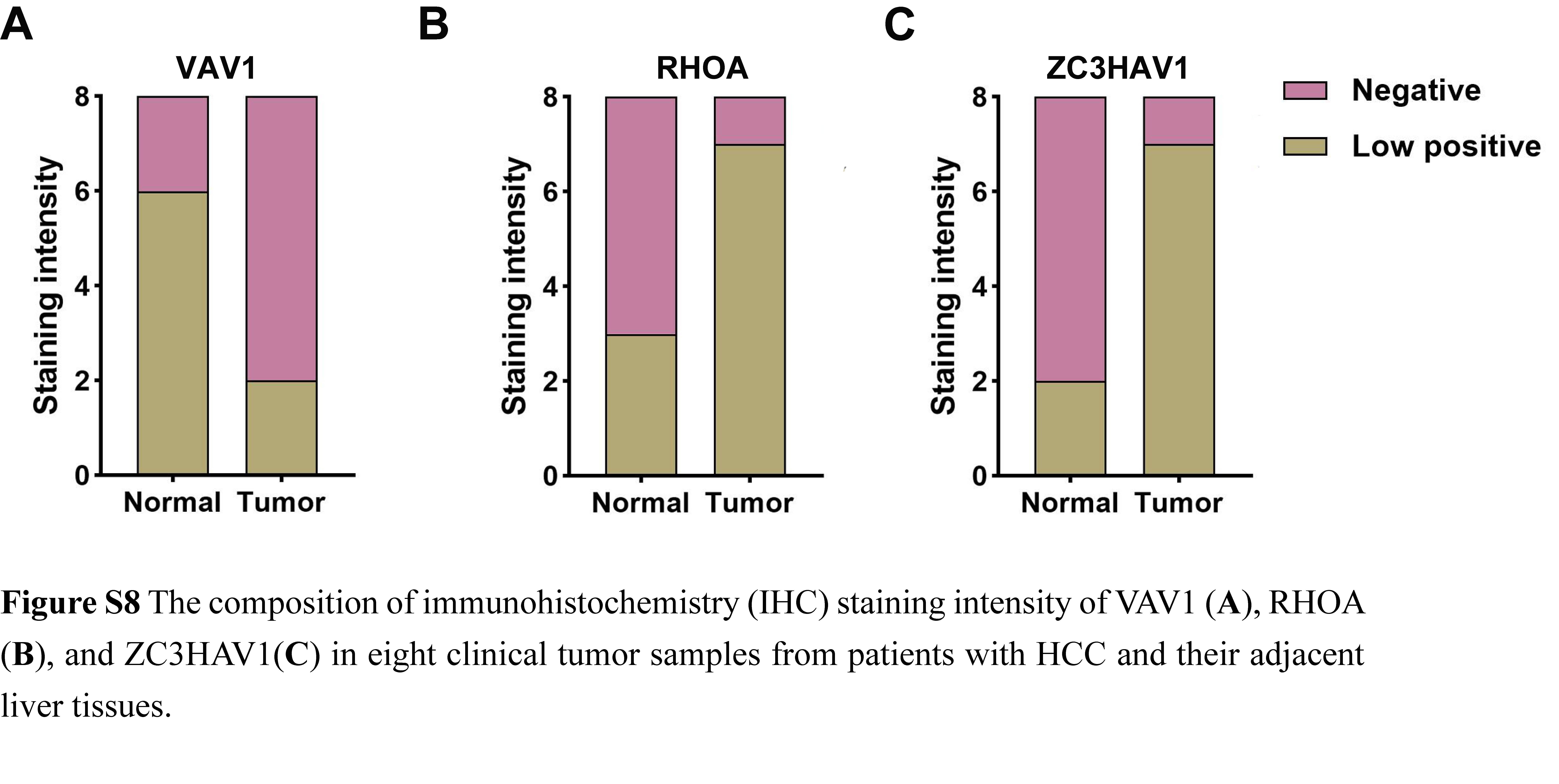

Supplement: Supplementary file 12 — Additional file 12: Figure S8. The composition of immunohistochemistry (IHC) staining intensity of VAV1 (A), RHOA (B), and ZC3HAV1(C) in eight clinical tumor samples from patients with HCC and their adjacent liver tissues. [file 12935_2022_2734_MOESM12_ESM.tif]
