# Supplementary material for: STING pathway contributes to the prognosis of hepatocellular carcinoma and identification of prognostic gene signatures correlated to tumor microenvironment
Source: Cancer Cell Int. 2022 Oct 12;22:314. doi: 10.1186/s12935-022-02734-4 (PMC9554977; doi:10.1186/s12935-022-02734-4)
Supplement: Supplementary file 10 — Additional file 10: Table S4. Pearson's correlation coefficient of overlapped co-expressed genes of NLRC3, STING1, TRIM21, TBK1, and XRCC6 from UALCAN database. [file 12935_2022_2734_MOESM10_ESM.docx]

**Table S4** Pearson's correlation coefficient of overlapped co-expressed genes of NLRC3, STING1, TRIM21, TBK1, and XRCC6 from UALCAN database.

| **Co-expressed Genes** | **Pearson correlation value** | | | | |
| --- | --- | --- | --- | --- | --- |
|  | **NLRC3** | **STING1** | **TRIM21** | **TBK1** | **XRCC6** |
| EVI2A | 0.85 | 0.57 | 0.5 | 0.35 | 0.31 |
| RUNX3 | 0.85 | 0.51 | 0.49 | 0.3 | 0.3 |
| SEMA4D | 0.83 | 0.56 | 0.5 | 0.38 | 0.31 |
| WIPF1 | 0.82 | 0.55 | 0.47 | 0.38 | 0.32 |
| TNFAIP8 | 0.78 | 0.51 | 0.56 | 0.43 | 0.3 |
| CLEC2D | 0.77 | 0.34 | 0.38 | 0.4 | 0.38 |
| NCKAP1L | 0.76 | 0.58 | 0.49 | 0.34 | 0.3 |
| CXCR4 | 0.75 | 0.5 | 0.42 | 0.37 | 0.31 |
| PTPN6 | 0.75 | 0.48 | 0.55 | 0.42 | 0.39 |
| TRAF1 | 0.75 | 0.41 | 0.49 | 0.43 | 0.3 |
| ZNF101 | 0.75 | 0.37 | 0.43 | 0.5 | 0.39 |
| STK10 | 0.73 | 0.61 | 0.56 | 0.44 | 0.38 |
| VAV1 | 0.73 | 0.58 | 0.44 | 0.32 | 0.31 |
| FMNL1 | 0.72 | 0.59 | 0.45 | 0.34 | 0.34 |
| DOK3 | 0.71 | 0.6 | 0.49 | 0.33 | 0.34 |
| LAPTM5 | 0.67 | 0.61 | 0.48 | 0.3 | 0.35 |
| ADCY7 | 0.66 | 0.63 | 0.49 | 0.39 | 0.38 |
| CRLF3 | 0.66 | 0.42 | 0.48 | 0.68 | 0.45 |
| PREX1 | 0.65 | 0.6 | 0.51 | 0.42 | 0.33 |
| SERPINB9 | 0.65 | 0.47 | 0.43 | 0.44 | 0.3 |
| STK4 | 0.65 | 0.41 | 0.5 | 0.69 | 0.48 |
| DENND2D | 0.65 | 0.4 | 0.43 | 0.36 | 0.31 |
| MSL3 | 0.64 | 0.56 | 0.56 | 0.6 | 0.48 |
| SYT11 | 0.64 | 0.56 | 0.41 | 0.4 | 0.36 |
| GMIP | 0.64 | 0.49 | 0.51 | 0.48 | 0.44 |
| MYCBP2 | 0.64 | 0.49 | 0.45 | 0.55 | 0.31 |
| ATM | 0.64 | 0.43 | 0.47 | 0.64 | 0.37 |
| ENTPD1 | 0.63 | 0.59 | 0.43 | 0.5 | 0.34 |
| CD83 | 0.63 | 0.39 | 0.42 | 0.37 | 0.37 |
| PLEKHA2 | 0.62 | 0.64 | 0.49 | 0.51 | 0.42 |
| SYK | 0.62 | 0.61 | 0.49 | 0.39 | 0.38 |
| CD86 | 0.62 | 0.56 | 0.51 | 0.35 | 0.37 |
| CYBB | 0.62 | 0.52 | 0.46 | 0.35 | 0.31 |
| PIP4K2A | 0.61 | 0.66 | 0.51 | 0.52 | 0.44 |
| FMNL3 | 0.61 | 0.57 | 0.51 | 0.46 | 0.43 |
| ST8SIA4 | 0.61 | 0.48 | 0.49 | 0.38 | 0.32 |
| DENND1C | 0.61 | 0.34 | 0.33 | 0.42 | 0.3 |
| FPR3 | 0.6 | 0.64 | 0.51 | 0.4 | 0.31 |
| APOBEC3C | 0.6 | 0.63 | 0.47 | 0.37 | 0.38 |
| APOB48R | 0.6 | 0.57 | 0.49 | 0.36 | 0.31 |
| ZNF267 | 0.6 | 0.47 | 0.6 | 0.7 | 0.44 |
| HCK | 0.59 | 0.57 | 0.46 | 0.32 | 0.33 |
| RILPL2 | 0.59 | 0.51 | 0.45 | 0.38 | 0.35 |
| CSK | 0.59 | 0.43 | 0.51 | 0.42 | 0.54 |
| C21orf91 | 0.59 | 0.41 | 0.48 | 0.51 | 0.33 |
| LOC374443 | 0.59 | 0.41 | 0.48 | 0.37 | 0.44 |
| SMCHD1 | 0.58 | 0.39 | 0.48 | 0.66 | 0.44 |
| SLC9A9 | 0.57 | 0.62 | 0.48 | 0.33 | 0.34 |
| ITGB2 | 0.56 | 0.5 | 0.44 | 0.3 | 0.3 |
| MBNL1 | 0.56 | 0.43 | 0.47 | 0.61 | 0.4 |
| SEMA4A | 0.56 | 0.42 | 0.4 | 0.3 | 0.41 |
| IFI16 | 0.55 | 0.55 | 0.51 | 0.35 | 0.35 |
| CLEC4A | 0.55 | 0.51 | 0.45 | 0.33 | 0.32 |
| PARP11 | 0.55 | 0.43 | 0.53 | 0.53 | 0.32 |
| FCHSD2 | 0.54 | 0.55 | 0.51 | 0.6 | 0.37 |
| ZBTB1 | 0.54 | 0.47 | 0.51 | 0.65 | 0.37 |
| PARP8 | 0.54 | 0.43 | 0.5 | 0.52 | 0.32 |
| ARAP2 | 0.54 | 0.41 | 0.52 | 0.54 | 0.3 |
| CDC42SE2 | 0.54 | 0.34 | 0.54 | 0.65 | 0.35 |
| PLEKHO2 | 0.53 | 0.72 | 0.47 | 0.31 | 0.36 |
| RASA3 | 0.53 | 0.56 | 0.38 | 0.35 | 0.32 |
| AMPD3 | 0.53 | 0.55 | 0.48 | 0.48 | 0.46 |
| ADRBK1 | 0.53 | 0.47 | 0.55 | 0.52 | 0.46 |
| PDE7A | 0.53 | 0.43 | 0.35 | 0.42 | 0.47 |
| PASK | 0.53 | 0.38 | 0.44 | 0.49 | 0.48 |
| MX2 | 0.53 | 0.32 | 0.49 | 0.32 | 0.32 |
| DOK1 | 0.52 | 0.65 | 0.48 | 0.36 | 0.49 |
| C3AR1 | 0.52 | 0.58 | 0.47 | 0.33 | 0.39 |
| GNB4 | 0.52 | 0.57 | 0.45 | 0.38 | 0.41 |
| ARL6IP5 | 0.52 | 0.55 | 0.5 | 0.53 | 0.39 |
| PTGER4 | 0.52 | 0.55 | 0.38 | 0.38 | 0.34 |
| TEP1 | 0.52 | 0.54 | 0.51 | 0.48 | 0.36 |
| RGS19 | 0.52 | 0.51 | 0.48 | 0.36 | 0.5 |
| MNDA | 0.52 | 0.5 | 0.46 | 0.39 | 0.31 |
| CLEC7A | 0.52 | 0.49 | 0.5 | 0.36 | 0.38 |
| SLC12A6 | 0.52 | 0.45 | 0.38 | 0.6 | 0.35 |
| STK17A | 0.52 | 0.34 | 0.47 | 0.52 | 0.52 |
| KIAA1949 | 0.51 | 0.51 | 0.53 | 0.48 | 0.48 |
| MOBKL2A | 0.51 | 0.5 | 0.4 | 0.4 | 0.49 |
| RELT | 0.51 | 0.47 | 0.51 | 0.54 | 0.51 |
| FNBP1 | 0.51 | 0.4 | 0.4 | 0.59 | 0.41 |
| DENND3 | 0.5 | 0.62 | 0.4 | 0.32 | 0.31 |
| BCAS4 | 0.5 | 0.55 | 0.42 | 0.31 | 0.48 |
| INPP4A | 0.5 | 0.53 | 0.46 | 0.56 | 0.41 |
| GIT2 | 0.5 | 0.47 | 0.52 | 0.74 | 0.49 |
| TRAFD1 | 0.5 | 0.36 | 0.63 | 0.65 | 0.52 |
| NOD1 | 0.49 | 0.77 | 0.48 | 0.42 | 0.3 |
| ARPC2 | 0.49 | 0.51 | 0.52 | 0.53 | 0.63 |
| IKZF4 | 0.49 | 0.5 | 0.47 | 0.7 | 0.4 |
| BMP2K | 0.49 | 0.44 | 0.43 | 0.55 | 0.38 |
| SGTB | 0.49 | 0.41 | 0.47 | 0.61 | 0.45 |
| LOC440354 | 0.49 | 0.36 | 0.37 | 0.56 | 0.31 |
| ZNF107 | 0.49 | 0.33 | 0.43 | 0.64 | 0.4 |
| TM6SF1 | 0.48 | 0.64 | 0.48 | 0.39 | 0.35 |
| CCND3 | 0.48 | 0.62 | 0.41 | 0.33 | 0.35 |
| LYN | 0.48 | 0.58 | 0.51 | 0.53 | 0.32 |
| NAGK | 0.48 | 0.54 | 0.49 | 0.36 | 0.53 |
| SNX6 | 0.48 | 0.53 | 0.46 | 0.66 | 0.52 |
| NECAP2 | 0.48 | 0.51 | 0.56 | 0.51 | 0.52 |
| ACTR3 | 0.48 | 0.5 | 0.55 | 0.67 | 0.56 |
| FRYL | 0.48 | 0.48 | 0.49 | 0.58 | 0.42 |
| PRDM2 | 0.48 | 0.47 | 0.46 | 0.56 | 0.38 |
| C14orf106 | 0.48 | 0.44 | 0.48 | 0.69 | 0.49 |
| PRKACB | 0.48 | 0.44 | 0.48 | 0.64 | 0.37 |
| JAK2 | 0.48 | 0.42 | 0.51 | 0.44 | 0.37 |
| RAB8B | 0.48 | 0.4 | 0.4 | 0.53 | 0.36 |
| IRAK4 | 0.48 | 0.39 | 0.48 | 0.74 | 0.45 |
| CCDC109B | 0.48 | 0.39 | 0.48 | 0.43 | 0.39 |
| NPAT | 0.48 | 0.38 | 0.51 | 0.71 | 0.48 |
| HIVEP2 | 0.48 | 0.36 | 0.37 | 0.62 | 0.36 |
| DCK | 0.48 | 0.34 | 0.49 | 0.69 | 0.44 |
| MYLIP | 0.47 | 0.73 | 0.38 | 0.44 | 0.38 |
| AXL | 0.47 | 0.72 | 0.41 | 0.32 | 0.32 |
| RAB31 | 0.47 | 0.69 | 0.4 | 0.37 | 0.41 |
| ADAP2 | 0.47 | 0.64 | 0.45 | 0.37 | 0.39 |
| IQGAP1 | 0.47 | 0.62 | 0.45 | 0.46 | 0.4 |
| PPP1R9B | 0.47 | 0.59 | 0.46 | 0.49 | 0.52 |
| EHD1 | 0.47 | 0.46 | 0.51 | 0.44 | 0.46 |
| ELF1 | 0.47 | 0.44 | 0.38 | 0.59 | 0.36 |
| TRIM34 | 0.47 | 0.43 | 0.73 | 0.58 | 0.37 |
| PPP1R12A | 0.47 | 0.42 | 0.44 | 0.79 | 0.47 |
| FCHSD1 | 0.47 | 0.4 | 0.38 | 0.32 | 0.35 |
| SETX | 0.47 | 0.39 | 0.47 | 0.69 | 0.37 |
| BAZ1A | 0.47 | 0.38 | 0.46 | 0.64 | 0.34 |
| IL27RA | 0.47 | 0.38 | 0.42 | 0.36 | 0.36 |
| ZRSR2 | 0.47 | 0.37 | 0.39 | 0.36 | 0.39 |
| SSH2 | 0.47 | 0.32 | 0.43 | 0.61 | 0.47 |
| ARHGAP27 | 0.46 | 0.57 | 0.48 | 0.42 | 0.33 |
| MAX | 0.46 | 0.53 | 0.54 | 0.52 | 0.43 |
| CORO7 | 0.46 | 0.5 | 0.5 | 0.44 | 0.42 |
| CBLB | 0.46 | 0.49 | 0.44 | 0.48 | 0.32 |
| ADAM8 | 0.46 | 0.49 | 0.38 | 0.36 | 0.36 |
| VPS13C | 0.46 | 0.48 | 0.46 | 0.62 | 0.32 |
| SLC4A7 | 0.46 | 0.43 | 0.44 | 0.58 | 0.39 |
| MOBKL1B | 0.46 | 0.42 | 0.53 | 0.74 | 0.41 |
| ACTR2 | 0.46 | 0.41 | 0.48 | 0.76 | 0.49 |
| DGKZ | 0.46 | 0.4 | 0.5 | 0.39 | 0.56 |
| IKBKE | 0.46 | 0.36 | 0.48 | 0.44 | 0.49 |
| CNN2 | 0.46 | 0.34 | 0.32 | 0.32 | 0.39 |
| IRF2 | 0.46 | 0.33 | 0.56 | 0.63 | 0.31 |
| ADPRH | 0.45 | 0.65 | 0.43 | 0.44 | 0.34 |
| RALGDS | 0.45 | 0.52 | 0.43 | 0.37 | 0.44 |
| KIAA0226 | 0.45 | 0.49 | 0.53 | 0.65 | 0.49 |
| TMED8 | 0.45 | 0.49 | 0.39 | 0.52 | 0.48 |
| KCTD11 | 0.45 | 0.48 | 0.38 | 0.46 | 0.41 |
| CBL | 0.45 | 0.45 | 0.48 | 0.61 | 0.48 |
| TRAF5 | 0.45 | 0.45 | 0.36 | 0.41 | 0.38 |
| SNAP23 | 0.45 | 0.43 | 0.49 | 0.67 | 0.42 |
| ZBTB24 | 0.45 | 0.34 | 0.43 | 0.6 | 0.45 |
| LOC388692 | 0.45 | 0.34 | 0.38 | 0.33 | 0.35 |
| KIF2A | 0.45 | 0.33 | 0.48 | 0.73 | 0.54 |
| DCP2 | 0.45 | 0.32 | 0.42 | 0.55 | 0.47 |
| ANKRD12 | 0.45 | 0.32 | 0.37 | 0.65 | 0.3 |
| ARHGAP18 | 0.44 | 0.59 | 0.32 | 0.49 | 0.43 |
| NSMAF | 0.44 | 0.54 | 0.45 | 0.53 | 0.5 |
| C15orf57 | 0.44 | 0.53 | 0.45 | 0.5 | 0.42 |
| SH3KBP1 | 0.44 | 0.53 | 0.42 | 0.38 | 0.43 |
| C12orf5 | 0.44 | 0.5 | 0.43 | 0.53 | 0.32 |
| WDR82 | 0.44 | 0.44 | 0.46 | 0.7 | 0.48 |
| SDCCAG1 | 0.44 | 0.44 | 0.41 | 0.61 | 0.44 |
| CCNG2 | 0.44 | 0.41 | 0.35 | 0.52 | 0.37 |
| NFKB1 | 0.44 | 0.39 | 0.48 | 0.64 | 0.4 |
| RFX7 | 0.44 | 0.38 | 0.37 | 0.59 | 0.39 |
| SCLT1 | 0.44 | 0.37 | 0.51 | 0.58 | 0.49 |
| CDK17 | 0.44 | 0.37 | 0.43 | 0.75 | 0.36 |
| PTPN4 | 0.44 | 0.35 | 0.44 | 0.53 | 0.42 |
| ZFC3H1 | 0.44 | 0.35 | 0.39 | 0.7 | 0.44 |
| SENP7 | 0.44 | 0.34 | 0.32 | 0.57 | 0.37 |
| EHBP1L1 | 0.43 | 0.65 | 0.52 | 0.5 | 0.49 |
| SDC3 | 0.43 | 0.62 | 0.45 | 0.33 | 0.43 |
| OBFC2A | 0.43 | 0.56 | 0.4 | 0.39 | 0.31 |
| FAM13B | 0.43 | 0.54 | 0.42 | 0.64 | 0.34 |
| TMX3 | 0.43 | 0.53 | 0.45 | 0.57 | 0.42 |
| PNRC2 | 0.43 | 0.51 | 0.45 | 0.62 | 0.36 |
| GNAI2 | 0.43 | 0.49 | 0.5 | 0.47 | 0.4 |
| RHOG | 0.43 | 0.47 | 0.54 | 0.31 | 0.52 |
| N4BP1 | 0.43 | 0.47 | 0.49 | 0.62 | 0.42 |
| MAN2B1 | 0.43 | 0.47 | 0.38 | 0.32 | 0.43 |
| SWAP70 | 0.43 | 0.43 | 0.6 | 0.71 | 0.42 |
| LINS1 | 0.43 | 0.43 | 0.42 | 0.52 | 0.37 |
| C12orf4 | 0.43 | 0.41 | 0.48 | 0.64 | 0.57 |
| BTN2A1 | 0.43 | 0.39 | 0.46 | 0.62 | 0.52 |
| HMGN4 | 0.43 | 0.38 | 0.5 | 0.59 | 0.56 |
| PTPN1 | 0.43 | 0.38 | 0.44 | 0.69 | 0.53 |
| CSGALNACT2 | 0.43 | 0.36 | 0.38 | 0.68 | 0.38 |
| PRKD3 | 0.43 | 0.35 | 0.46 | 0.7 | 0.49 |
| UBE2J1 | 0.43 | 0.34 | 0.39 | 0.64 | 0.43 |
| CCDC66 | 0.43 | 0.34 | 0.39 | 0.61 | 0.44 |
| STIM2 | 0.43 | 0.32 | 0.51 | 0.62 | 0.41 |
| NIN | 0.43 | 0.31 | 0.42 | 0.55 | 0.35 |
| ZNF708 | 0.43 | 0.3 | 0.31 | 0.61 | 0.41 |
| MOBKL2C | 0.42 | 0.6 | 0.58 | 0.47 | 0.37 |
| APH1B | 0.42 | 0.57 | 0.35 | 0.44 | 0.37 |
| ELMO2 | 0.42 | 0.55 | 0.47 | 0.63 | 0.53 |
| RAP2B | 0.42 | 0.54 | 0.5 | 0.5 | 0.51 |
| YWHAB | 0.42 | 0.46 | 0.51 | 0.66 | 0.56 |
| ZZEF1 | 0.42 | 0.44 | 0.35 | 0.57 | 0.33 |
| GRINL1A | 0.42 | 0.43 | 0.37 | 0.62 | 0.44 |
| FUT8 | 0.42 | 0.43 | 0.37 | 0.4 | 0.48 |
| CBFB | 0.42 | 0.42 | 0.44 | 0.63 | 0.5 |
| AKAP11 | 0.42 | 0.42 | 0.32 | 0.65 | 0.3 |
| WDFY1 | 0.42 | 0.41 | 0.39 | 0.66 | 0.47 |
| MYO9B | 0.42 | 0.41 | 0.38 | 0.36 | 0.48 |
| RECQL | 0.42 | 0.4 | 0.47 | 0.73 | 0.5 |
| PAPOLG | 0.42 | 0.4 | 0.46 | 0.71 | 0.48 |
| UVRAG | 0.42 | 0.39 | 0.57 | 0.74 | 0.44 |
| HNRPLL | 0.42 | 0.39 | 0.51 | 0.75 | 0.61 |
| CHMP7 | 0.42 | 0.37 | 0.39 | 0.54 | 0.35 |
| PPP2R3C | 0.42 | 0.37 | 0.37 | 0.49 | 0.52 |
| C7orf23 | 0.42 | 0.35 | 0.45 | 0.42 | 0.35 |
| TRAF3 | 0.42 | 0.32 | 0.53 | 0.56 | 0.4 |
| FAM53B | 0.41 | 0.62 | 0.35 | 0.34 | 0.4 |
| HEG1 | 0.41 | 0.49 | 0.46 | 0.51 | 0.35 |
| SEC14L1 | 0.41 | 0.48 | 0.37 | 0.53 | 0.46 |
| LIMA1 | 0.41 | 0.46 | 0.5 | 0.54 | 0.44 |
| FGFR1OP2 | 0.41 | 0.46 | 0.46 | 0.65 | 0.53 |
| ACAP2 | 0.41 | 0.45 | 0.45 | 0.75 | 0.44 |
| UBR1 | 0.41 | 0.42 | 0.45 | 0.64 | 0.37 |
| CHST12 | 0.41 | 0.42 | 0.39 | 0.31 | 0.53 |
| RGS1 | 0.41 | 0.42 | 0.37 | 0.32 | 0.3 |
| ZNF589 | 0.41 | 0.41 | 0.33 | 0.41 | 0.46 |
| ELF4 | 0.41 | 0.4 | 0.46 | 0.5 | 0.43 |
| UBLCP1 | 0.41 | 0.39 | 0.45 | 0.67 | 0.42 |
| CREB1 | 0.41 | 0.39 | 0.44 | 0.7 | 0.51 |
| PCYOX1L | 0.41 | 0.39 | 0.42 | 0.44 | 0.43 |
| UBE2D1 | 0.41 | 0.39 | 0.39 | 0.59 | 0.49 |
| CASP4 | 0.41 | 0.38 | 0.53 | 0.44 | 0.38 |
| DDX6 | 0.41 | 0.37 | 0.43 | 0.68 | 0.42 |
| ZNF641 | 0.41 | 0.37 | 0.4 | 0.7 | 0.32 |
| BOD1L | 0.41 | 0.37 | 0.39 | 0.68 | 0.44 |
| SMARCA5 | 0.41 | 0.35 | 0.49 | 0.67 | 0.44 |
| CNOT8 | 0.41 | 0.35 | 0.46 | 0.66 | 0.44 |
| CHD2 | 0.41 | 0.34 | 0.37 | 0.6 | 0.43 |
| CFLAR | 0.41 | 0.32 | 0.44 | 0.62 | 0.47 |
| ADA | 0.41 | 0.31 | 0.45 | 0.32 | 0.46 |
| TINF2 | 0.41 | 0.3 | 0.45 | 0.55 | 0.44 |
| PML | 0.4 | 0.55 | 0.6 | 0.45 | 0.46 |
| SPRED1 | 0.4 | 0.54 | 0.44 | 0.53 | 0.45 |
| MKL1 | 0.4 | 0.52 | 0.43 | 0.46 | 0.63 |
| SGK269 | 0.4 | 0.52 | 0.33 | 0.53 | 0.35 |
| SLC7A7 | 0.4 | 0.5 | 0.47 | 0.3 | 0.36 |
| RB1 | 0.4 | 0.5 | 0.32 | 0.53 | 0.34 |
| BNIP2 | 0.4 | 0.45 | 0.36 | 0.53 | 0.3 |
| MAP3K3 | 0.4 | 0.44 | 0.39 | 0.61 | 0.46 |
| CRTC3 | 0.4 | 0.43 | 0.37 | 0.57 | 0.44 |
| PAN3 | 0.4 | 0.43 | 0.36 | 0.59 | 0.44 |
| PPM1M | 0.4 | 0.42 | 0.4 | 0.31 | 0.36 |
| SPTLC2 | 0.4 | 0.41 | 0.43 | 0.58 | 0.38 |
| SAMD8 | 0.4 | 0.4 | 0.35 | 0.68 | 0.38 |
| SMG1 | 0.4 | 0.38 | 0.45 | 0.66 | 0.37 |
| GCC2 | 0.4 | 0.37 | 0.35 | 0.62 | 0.41 |
| NDST2 | 0.4 | 0.37 | 0.32 | 0.47 | 0.48 |
| PRKX | 0.4 | 0.36 | 0.31 | 0.35 | 0.38 |
| DYRK2 | 0.4 | 0.35 | 0.48 | 0.69 | 0.54 |
| ROCK1 | 0.4 | 0.35 | 0.43 | 0.76 | 0.4 |
| PAPD4 | 0.4 | 0.35 | 0.39 | 0.68 | 0.31 |
| LOC284441 | 0.4 | 0.34 | 0.4 | 0.73 | 0.37 |
| C11orf46 | 0.4 | 0.31 | 0.48 | 0.6 | 0.41 |
| FAM65A | 0.39 | 0.61 | 0.43 | 0.5 | 0.44 |
| TLE4 | 0.39 | 0.59 | 0.35 | 0.36 | 0.37 |
| STX7 | 0.39 | 0.53 | 0.36 | 0.61 | 0.43 |
| ZBTB4 | 0.39 | 0.52 | 0.38 | 0.49 | 0.38 |
| C3orf64 | 0.39 | 0.5 | 0.47 | 0.54 | 0.43 |
| ADAMTS7 | 0.39 | 0.5 | 0.31 | 0.37 | 0.36 |
| RAP1B | 0.39 | 0.47 | 0.47 | 0.68 | 0.47 |
| LGALS9 | 0.39 | 0.45 | 0.45 | 0.37 | 0.36 |
| FAM102B | 0.39 | 0.43 | 0.44 | 0.56 | 0.41 |
| SMAD7 | 0.39 | 0.43 | 0.35 | 0.43 | 0.38 |
| C17orf85 | 0.39 | 0.42 | 0.35 | 0.62 | 0.43 |
| ZNFX1 | 0.39 | 0.41 | 0.61 | 0.6 | 0.34 |
| FAM48A | 0.39 | 0.41 | 0.36 | 0.57 | 0.54 |
| ARF6 | 0.39 | 0.39 | 0.44 | 0.58 | 0.44 |
| MINK1 | 0.39 | 0.39 | 0.4 | 0.57 | 0.49 |
| LRCH4 | 0.39 | 0.39 | 0.3 | 0.42 | 0.37 |
| CASP10 | 0.39 | 0.38 | 0.48 | 0.5 | 0.38 |
| KIAA0430 | 0.39 | 0.38 | 0.43 | 0.62 | 0.33 |
| C14orf118 | 0.39 | 0.38 | 0.42 | 0.69 | 0.45 |
| ATP10D | 0.39 | 0.35 | 0.42 | 0.58 | 0.33 |
| KIAA0586 | 0.39 | 0.34 | 0.39 | 0.64 | 0.4 |
| PRKRIR | 0.39 | 0.32 | 0.52 | 0.67 | 0.44 |
| CGGBP1 | 0.39 | 0.31 | 0.44 | 0.71 | 0.47 |
| TRAPPC10 | 0.39 | 0.31 | 0.4 | 0.62 | 0.38 |
| SYNRG | 0.39 | 0.3 | 0.38 | 0.7 | 0.44 |
| SNN | 0.38 | 0.61 | 0.43 | 0.47 | 0.33 |
| TWSG1 | 0.38 | 0.59 | 0.47 | 0.57 | 0.42 |
| LUZP1 | 0.38 | 0.57 | 0.45 | 0.61 | 0.44 |
| AFAP1 | 0.38 | 0.56 | 0.33 | 0.33 | 0.34 |
| RPS6KA4 | 0.38 | 0.55 | 0.52 | 0.46 | 0.54 |
| NAV1 | 0.38 | 0.55 | 0.39 | 0.36 | 0.38 |
| PPP1R3D | 0.38 | 0.55 | 0.36 | 0.44 | 0.33 |
| ARHGAP17 | 0.38 | 0.53 | 0.53 | 0.56 | 0.4 |
| RAB2B | 0.38 | 0.52 | 0.38 | 0.56 | 0.36 |
| KCTD10 | 0.38 | 0.5 | 0.41 | 0.6 | 0.51 |
| SUSD1 | 0.38 | 0.48 | 0.36 | 0.55 | 0.55 |
| RSBN1 | 0.38 | 0.47 | 0.39 | 0.59 | 0.4 |
| FKBP15 | 0.38 | 0.46 | 0.53 | 0.66 | 0.47 |
| BTBD1 | 0.38 | 0.45 | 0.41 | 0.58 | 0.41 |
| CAP1 | 0.38 | 0.44 | 0.56 | 0.67 | 0.53 |
| CALM1 | 0.38 | 0.44 | 0.37 | 0.37 | 0.34 |
| ETV6 | 0.38 | 0.43 | 0.56 | 0.54 | 0.45 |
| PAFAH1B1 | 0.38 | 0.43 | 0.36 | 0.59 | 0.44 |
| C7orf60 | 0.38 | 0.41 | 0.42 | 0.55 | 0.59 |
| UBA3 | 0.38 | 0.4 | 0.51 | 0.72 | 0.56 |
| PHTF2 | 0.38 | 0.4 | 0.46 | 0.64 | 0.5 |
| LDB1 | 0.38 | 0.4 | 0.37 | 0.58 | 0.53 |
| CCNDBP1 | 0.38 | 0.39 | 0.45 | 0.56 | 0.38 |
| ATF7IP | 0.38 | 0.39 | 0.44 | 0.64 | 0.41 |
| MLL | 0.38 | 0.39 | 0.38 | 0.51 | 0.47 |
| ZNF211 | 0.38 | 0.39 | 0.34 | 0.42 | 0.39 |
| PIK3C3 | 0.38 | 0.38 | 0.46 | 0.69 | 0.59 |
| C14orf43 | 0.38 | 0.38 | 0.4 | 0.62 | 0.36 |
| ADPGK | 0.38 | 0.37 | 0.44 | 0.44 | 0.54 |
| RAPGEF1 | 0.38 | 0.37 | 0.41 | 0.64 | 0.43 |
| RBM25 | 0.38 | 0.37 | 0.4 | 0.68 | 0.43 |
| MORF4L1 | 0.38 | 0.37 | 0.4 | 0.55 | 0.66 |
| ALKBH8 | 0.38 | 0.37 | 0.4 | 0.54 | 0.43 |
| DMTF1 | 0.38 | 0.37 | 0.37 | 0.62 | 0.42 |
| C3orf38 | 0.38 | 0.36 | 0.54 | 0.7 | 0.52 |
| VOPP1 | 0.38 | 0.36 | 0.44 | 0.37 | 0.52 |
| YTHDC1 | 0.38 | 0.35 | 0.42 | 0.66 | 0.47 |
| ZBTB6 | 0.38 | 0.35 | 0.42 | 0.65 | 0.42 |
| INPP5B | 0.38 | 0.35 | 0.39 | 0.47 | 0.31 |
| KIAA2026 | 0.38 | 0.35 | 0.38 | 0.56 | 0.47 |
| SYNJ1 | 0.38 | 0.34 | 0.4 | 0.7 | 0.4 |
| EPC2 | 0.38 | 0.34 | 0.37 | 0.68 | 0.46 |
| FAM49A | 0.38 | 0.34 | 0.32 | 0.37 | 0.3 |
| DDX3X | 0.38 | 0.33 | 0.41 | 0.76 | 0.38 |
| PRPF38B | 0.38 | 0.33 | 0.4 | 0.63 | 0.46 |
| MDM1 | 0.38 | 0.32 | 0.46 | 0.74 | 0.43 |
| ZNF136 | 0.38 | 0.32 | 0.38 | 0.61 | 0.35 |
| MED13L | 0.38 | 0.32 | 0.36 | 0.74 | 0.35 |
| CENPC1 | 0.38 | 0.31 | 0.38 | 0.59 | 0.3 |
| MAP3K1 | 0.38 | 0.31 | 0.38 | 0.55 | 0.42 |
| MSL2 | 0.38 | 0.3 | 0.4 | 0.65 | 0.45 |
| MAP7D1 | 0.37 | 0.59 | 0.46 | 0.36 | 0.48 |
| SPARC | 0.37 | 0.59 | 0.3 | 0.32 | 0.3 |
| TLN1 | 0.37 | 0.5 | 0.48 | 0.65 | 0.37 |
| SYNGAP1 | 0.37 | 0.49 | 0.34 | 0.44 | 0.38 |
| IKBKB | 0.37 | 0.48 | 0.39 | 0.55 | 0.41 |
| ANKFY1 | 0.37 | 0.45 | 0.41 | 0.59 | 0.31 |
| M6PR | 0.37 | 0.44 | 0.44 | 0.61 | 0.54 |
| CAMTA2 | 0.37 | 0.44 | 0.33 | 0.41 | 0.33 |
| PHC1 | 0.37 | 0.44 | 0.31 | 0.37 | 0.48 |
| GCA | 0.37 | 0.43 | 0.43 | 0.52 | 0.45 |
| SFT2D1 | 0.37 | 0.43 | 0.43 | 0.4 | 0.6 |
| NXF1 | 0.37 | 0.43 | 0.38 | 0.56 | 0.57 |
| VPS4B | 0.37 | 0.42 | 0.47 | 0.7 | 0.36 |
| CSF2RA | 0.37 | 0.4 | 0.33 | 0.31 | 0.34 |
| ZDHHC21 | 0.37 | 0.39 | 0.44 | 0.69 | 0.39 |
| MTA2 | 0.37 | 0.38 | 0.59 | 0.65 | 0.55 |
| ADO | 0.37 | 0.38 | 0.37 | 0.62 | 0.58 |
| PLDN | 0.37 | 0.37 | 0.37 | 0.65 | 0.42 |
| ME2 | 0.37 | 0.36 | 0.52 | 0.69 | 0.44 |
| DFFB | 0.37 | 0.36 | 0.37 | 0.51 | 0.49 |
| ATP8B2 | 0.37 | 0.36 | 0.31 | 0.45 | 0.39 |
| OFD1 | 0.37 | 0.35 | 0.36 | 0.49 | 0.45 |
| MBD2 | 0.37 | 0.34 | 0.48 | 0.6 | 0.55 |
| CCDC112 | 0.37 | 0.34 | 0.41 | 0.47 | 0.43 |
| USP34 | 0.37 | 0.34 | 0.38 | 0.59 | 0.55 |
| ZNF41 | 0.37 | 0.34 | 0.37 | 0.66 | 0.33 |
| MLL5 | 0.37 | 0.34 | 0.35 | 0.65 | 0.39 |
| MYST3 | 0.37 | 0.34 | 0.34 | 0.66 | 0.38 |
| METTL4 | 0.37 | 0.33 | 0.47 | 0.58 | 0.55 |
| RBM12 | 0.37 | 0.33 | 0.43 | 0.73 | 0.6 |
| SMURF2 | 0.37 | 0.33 | 0.39 | 0.59 | 0.51 |
| ATRX | 0.37 | 0.32 | 0.38 | 0.71 | 0.4 |
| CDKN2AIP | 0.37 | 0.31 | 0.37 | 0.64 | 0.33 |
| PIKFYVE | 0.37 | 0.31 | 0.34 | 0.7 | 0.34 |
| ZDHHC17 | 0.37 | 0.31 | 0.33 | 0.74 | 0.4 |
| PTPN2 | 0.37 | 0.3 | 0.42 | 0.46 | 0.5 |
| MSR1 | 0.36 | 0.58 | 0.47 | 0.4 | 0.39 |
| KLC1 | 0.36 | 0.55 | 0.35 | 0.36 | 0.46 |
| SNTB2 | 0.36 | 0.53 | 0.43 | 0.57 | 0.4 |
| DUSP7 | 0.36 | 0.52 | 0.41 | 0.53 | 0.48 |
| CD97 | 0.36 | 0.51 | 0.36 | 0.35 | 0.37 |
| B3GNT2 | 0.36 | 0.48 | 0.42 | 0.57 | 0.36 |
| VASH1 | 0.36 | 0.47 | 0.43 | 0.39 | 0.42 |
| GNPDA2 | 0.36 | 0.47 | 0.37 | 0.55 | 0.44 |
| SKAP2 | 0.36 | 0.45 | 0.4 | 0.59 | 0.36 |
| MFSD1 | 0.36 | 0.43 | 0.46 | 0.63 | 0.35 |
| TRA2A | 0.36 | 0.43 | 0.41 | 0.63 | 0.44 |
| MTMR9 | 0.36 | 0.43 | 0.35 | 0.62 | 0.31 |
| SOCS4 | 0.36 | 0.42 | 0.44 | 0.66 | 0.39 |
| ODF2L | 0.36 | 0.42 | 0.44 | 0.42 | 0.4 |
| RNF145 | 0.36 | 0.41 | 0.41 | 0.55 | 0.43 |
| WDR1 | 0.36 | 0.41 | 0.4 | 0.53 | 0.55 |
| BBX | 0.36 | 0.4 | 0.47 | 0.7 | 0.38 |
| CAB39 | 0.36 | 0.4 | 0.45 | 0.71 | 0.46 |
| ZNF484 | 0.36 | 0.4 | 0.43 | 0.65 | 0.4 |
| DCP1A | 0.36 | 0.39 | 0.42 | 0.68 | 0.48 |
| ARAP1 | 0.36 | 0.38 | 0.43 | 0.41 | 0.53 |
| DBNL | 0.36 | 0.38 | 0.39 | 0.36 | 0.47 |
| RSF1 | 0.36 | 0.37 | 0.44 | 0.73 | 0.41 |
| LUZP6 | 0.36 | 0.37 | 0.44 | 0.71 | 0.48 |
| POLR2A | 0.36 | 0.37 | 0.42 | 0.68 | 0.45 |
| ZNF20 | 0.36 | 0.37 | 0.42 | 0.51 | 0.49 |
| PLSCR1 | 0.36 | 0.36 | 0.54 | 0.49 | 0.38 |
| DUSP11 | 0.36 | 0.36 | 0.48 | 0.64 | 0.58 |
| YLPM1 | 0.36 | 0.36 | 0.43 | 0.66 | 0.48 |
| SON | 0.36 | 0.36 | 0.42 | 0.65 | 0.47 |
| TMX1 | 0.36 | 0.35 | 0.5 | 0.64 | 0.41 |
| PTPRA | 0.36 | 0.35 | 0.44 | 0.58 | 0.53 |
| DYRK1A | 0.36 | 0.35 | 0.4 | 0.67 | 0.42 |
| CTSC | 0.36 | 0.35 | 0.4 | 0.38 | 0.46 |
| MGAT4A | 0.36 | 0.35 | 0.36 | 0.5 | 0.46 |
| CTCF | 0.36 | 0.33 | 0.49 | 0.68 | 0.54 |
| CXorf38 | 0.36 | 0.33 | 0.46 | 0.56 | 0.34 |
| PHF15 | 0.36 | 0.33 | 0.41 | 0.46 | 0.42 |
| PHC3 | 0.36 | 0.33 | 0.4 | 0.71 | 0.38 |
| TET2 | 0.36 | 0.33 | 0.36 | 0.57 | 0.34 |
| DPP8 | 0.36 | 0.33 | 0.34 | 0.68 | 0.36 |
| CHD8 | 0.36 | 0.32 | 0.44 | 0.66 | 0.54 |
| CDC42SE1 | 0.36 | 0.32 | 0.44 | 0.63 | 0.45 |
| RNF111 | 0.36 | 0.31 | 0.42 | 0.68 | 0.46 |
| AP4E1 | 0.36 | 0.31 | 0.41 | 0.71 | 0.41 |
| NEDD1 | 0.36 | 0.3 | 0.49 | 0.81 | 0.52 |
| CYB5R4 | 0.36 | 0.3 | 0.43 | 0.61 | 0.34 |
| ZBTB49 | 0.36 | 0.3 | 0.41 | 0.54 | 0.48 |
| MSN | 0.35 | 0.53 | 0.48 | 0.52 | 0.35 |
| FCGR2A | 0.35 | 0.53 | 0.45 | 0.34 | 0.38 |
| AGTPBP1 | 0.35 | 0.53 | 0.41 | 0.57 | 0.32 |
| PIP5K1C | 0.35 | 0.53 | 0.4 | 0.44 | 0.43 |
| MOBKL1A | 0.35 | 0.53 | 0.37 | 0.57 | 0.38 |
| EPS15 | 0.35 | 0.5 | 0.52 | 0.73 | 0.35 |
| PRMT2 | 0.35 | 0.5 | 0.33 | 0.35 | 0.52 |
| TMEM87B | 0.35 | 0.49 | 0.41 | 0.51 | 0.35 |
| DMXL2 | 0.35 | 0.48 | 0.38 | 0.51 | 0.32 |
| FBXO34 | 0.35 | 0.46 | 0.43 | 0.54 | 0.38 |
| OSBPL7 | 0.35 | 0.46 | 0.4 | 0.37 | 0.46 |
| NR2F2 | 0.35 | 0.46 | 0.32 | 0.43 | 0.33 |
| DUSP18 | 0.35 | 0.45 | 0.46 | 0.55 | 0.49 |
| HIATL1 | 0.35 | 0.45 | 0.45 | 0.67 | 0.51 |
| RALB | 0.35 | 0.45 | 0.45 | 0.5 | 0.52 |
| PACS1 | 0.35 | 0.45 | 0.4 | 0.37 | 0.44 |
| ZFYVE26 | 0.35 | 0.44 | 0.47 | 0.62 | 0.44 |
| BMPR2 | 0.35 | 0.44 | 0.39 | 0.63 | 0.34 |
| TGFBRAP1 | 0.35 | 0.43 | 0.36 | 0.65 | 0.4 |
| TERF2IP | 0.35 | 0.43 | 0.3 | 0.44 | 0.34 |
| WDR47 | 0.35 | 0.42 | 0.46 | 0.66 | 0.42 |
| GTPBP8 | 0.35 | 0.42 | 0.45 | 0.54 | 0.52 |
| HDAC7 | 0.35 | 0.42 | 0.36 | 0.36 | 0.47 |
| DENND5A | 0.35 | 0.41 | 0.59 | 0.71 | 0.5 |
| PPT1 | 0.35 | 0.4 | 0.51 | 0.49 | 0.54 |
| SSH1 | 0.35 | 0.39 | 0.48 | 0.74 | 0.36 |
| VPS8 | 0.35 | 0.39 | 0.46 | 0.65 | 0.57 |
| UTP3 | 0.35 | 0.39 | 0.46 | 0.56 | 0.39 |
| C13orf23 | 0.35 | 0.39 | 0.44 | 0.64 | 0.55 |
| ZNF17 | 0.35 | 0.38 | 0.42 | 0.57 | 0.47 |
| KIAA0247 | 0.35 | 0.38 | 0.38 | 0.51 | 0.32 |
| AKAP10 | 0.35 | 0.38 | 0.37 | 0.65 | 0.44 |
| CHIC2 | 0.35 | 0.38 | 0.37 | 0.49 | 0.4 |
| DIP2A | 0.35 | 0.38 | 0.34 | 0.55 | 0.42 |
| XPO6 | 0.35 | 0.37 | 0.55 | 0.63 | 0.58 |
| MED17 | 0.35 | 0.37 | 0.47 | 0.65 | 0.57 |
| PXK | 0.35 | 0.37 | 0.43 | 0.66 | 0.31 |
| INO80 | 0.35 | 0.36 | 0.48 | 0.66 | 0.49 |
| ZNF597 | 0.35 | 0.36 | 0.45 | 0.55 | 0.37 |
| CCNI | 0.35 | 0.36 | 0.38 | 0.59 | 0.38 |
| ZC3HAV1 | 0.35 | 0.34 | 0.57 | 0.58 | 0.38 |
| KIAA1468 | 0.35 | 0.34 | 0.44 | 0.72 | 0.45 |
| TMF1 | 0.35 | 0.34 | 0.41 | 0.79 | 0.4 |
| TANK | 0.35 | 0.34 | 0.38 | 0.61 | 0.35 |
| ADAM10 | 0.35 | 0.33 | 0.4 | 0.62 | 0.36 |
| ATR | 0.35 | 0.32 | 0.44 | 0.73 | 0.46 |
| PPIL4 | 0.35 | 0.32 | 0.4 | 0.68 | 0.45 |
| PCF11 | 0.35 | 0.32 | 0.34 | 0.56 | 0.35 |
| LARP7 | 0.35 | 0.31 | 0.45 | 0.6 | 0.34 |
| ATP11B | 0.35 | 0.31 | 0.43 | 0.65 | 0.42 |
| RPIA | 0.35 | 0.31 | 0.37 | 0.43 | 0.63 |
| BCLAF1 | 0.35 | 0.31 | 0.36 | 0.7 | 0.48 |
| MPHOSPH8 | 0.35 | 0.31 | 0.34 | 0.66 | 0.36 |
| RCHY1 | 0.35 | 0.3 | 0.41 | 0.59 | 0.35 |
| TANC1 | 0.34 | 0.67 | 0.42 | 0.53 | 0.37 |
| ZFP106 | 0.34 | 0.6 | 0.39 | 0.55 | 0.38 |
| RGL1 | 0.34 | 0.6 | 0.34 | 0.45 | 0.33 |
| RARG | 0.34 | 0.55 | 0.39 | 0.35 | 0.39 |
| ATP6V1B2 | 0.34 | 0.49 | 0.41 | 0.58 | 0.32 |
| BTBD10 | 0.34 | 0.48 | 0.57 | 0.68 | 0.54 |
| METTL9 | 0.34 | 0.47 | 0.42 | 0.47 | 0.56 |
| MAP4K5 | 0.34 | 0.47 | 0.38 | 0.59 | 0.44 |
| BICD2 | 0.34 | 0.47 | 0.33 | 0.6 | 0.48 |
| MED15 | 0.34 | 0.46 | 0.4 | 0.46 | 0.71 |
| TPM4 | 0.34 | 0.46 | 0.4 | 0.43 | 0.49 |
| PELI1 | 0.34 | 0.45 | 0.35 | 0.5 | 0.37 |
| FIG4 | 0.34 | 0.42 | 0.41 | 0.57 | 0.49 |
| JAK1 | 0.34 | 0.39 | 0.49 | 0.67 | 0.39 |
| MAPRE1 | 0.34 | 0.39 | 0.41 | 0.56 | 0.64 |
| SOS2 | 0.34 | 0.39 | 0.36 | 0.57 | 0.3 |
| ATP13A2 | 0.34 | 0.38 | 0.46 | 0.34 | 0.55 |
| ATP7A | 0.34 | 0.38 | 0.45 | 0.66 | 0.48 |
| STARD3NL | 0.34 | 0.38 | 0.42 | 0.54 | 0.6 |
| NUDT21 | 0.34 | 0.37 | 0.44 | 0.68 | 0.52 |
| DCTN5 | 0.34 | 0.37 | 0.41 | 0.48 | 0.55 |
| SLC7A6 | 0.34 | 0.37 | 0.37 | 0.47 | 0.45 |
| WHAMM | 0.34 | 0.37 | 0.34 | 0.53 | 0.32 |
| NEURL4 | 0.34 | 0.37 | 0.34 | 0.46 | 0.5 |
| FAM98B | 0.34 | 0.36 | 0.47 | 0.7 | 0.55 |
| NIPA2 | 0.34 | 0.36 | 0.45 | 0.61 | 0.53 |
| DDX5 | 0.34 | 0.36 | 0.42 | 0.72 | 0.39 |
| TMEM206 | 0.34 | 0.36 | 0.41 | 0.55 | 0.52 |
| RAB35 | 0.34 | 0.35 | 0.5 | 0.71 | 0.58 |
| PTPN9 | 0.34 | 0.35 | 0.4 | 0.57 | 0.44 |
| IDS | 0.34 | 0.35 | 0.39 | 0.47 | 0.37 |
| ORAI2 | 0.34 | 0.35 | 0.37 | 0.32 | 0.45 |
| KBTBD2 | 0.34 | 0.33 | 0.47 | 0.74 | 0.5 |
| MEX3C | 0.34 | 0.33 | 0.4 | 0.65 | 0.51 |
| ARID1B | 0.34 | 0.33 | 0.39 | 0.67 | 0.45 |
| RANBP2 | 0.34 | 0.33 | 0.36 | 0.71 | 0.43 |
| MGA | 0.34 | 0.33 | 0.33 | 0.62 | 0.45 |
| C3orf63 | 0.34 | 0.32 | 0.44 | 0.73 | 0.47 |
| TRIP12 | 0.34 | 0.32 | 0.43 | 0.72 | 0.51 |
| RTN1 | 0.34 | 0.32 | 0.4 | 0.36 | 0.37 |
| CLDND1 | 0.34 | 0.32 | 0.39 | 0.63 | 0.46 |
| INO80D | 0.34 | 0.32 | 0.32 | 0.6 | 0.38 |
| C2CD3 | 0.34 | 0.31 | 0.45 | 0.65 | 0.53 |
| SLTM | 0.34 | 0.31 | 0.42 | 0.7 | 0.47 |
| FNBP4 | 0.34 | 0.31 | 0.4 | 0.62 | 0.45 |
| LRRC37B | 0.34 | 0.31 | 0.39 | 0.6 | 0.45 |
| RGPD4 | 0.34 | 0.31 | 0.36 | 0.65 | 0.43 |
| TRIM38 | 0.34 | 0.3 | 0.49 | 0.5 | 0.3 |
| NFE2L3 | 0.34 | 0.3 | 0.48 | 0.43 | 0.47 |
| SF3B1 | 0.34 | 0.3 | 0.39 | 0.73 | 0.47 |
| HNRNPH3 | 0.34 | 0.3 | 0.39 | 0.62 | 0.59 |
| SYDE1 | 0.33 | 0.63 | 0.36 | 0.35 | 0.44 |
| GYG1 | 0.33 | 0.57 | 0.36 | 0.48 | 0.52 |
| ZNF532 | 0.33 | 0.54 | 0.44 | 0.45 | 0.49 |
| EXOC1 | 0.33 | 0.52 | 0.47 | 0.62 | 0.45 |
| TMEM165 | 0.33 | 0.5 | 0.45 | 0.57 | 0.49 |
| CMTM1 | 0.33 | 0.49 | 0.42 | 0.48 | 0.51 |
| RUNX1 | 0.33 | 0.48 | 0.36 | 0.4 | 0.44 |
| ADAM17 | 0.33 | 0.47 | 0.49 | 0.61 | 0.5 |
| LDOC1L | 0.33 | 0.47 | 0.34 | 0.39 | 0.51 |
| PNMA1 | 0.33 | 0.47 | 0.33 | 0.45 | 0.54 |
| PCSK7 | 0.33 | 0.44 | 0.47 | 0.49 | 0.39 |
| ACTB | 0.33 | 0.42 | 0.4 | 0.33 | 0.48 |
| MIER1 | 0.33 | 0.41 | 0.46 | 0.67 | 0.34 |
| MACF1 | 0.33 | 0.39 | 0.45 | 0.48 | 0.44 |
| TMEM188 | 0.33 | 0.39 | 0.44 | 0.65 | 0.37 |
| RAB3GAP1 | 0.33 | 0.39 | 0.42 | 0.72 | 0.5 |
| DHX15 | 0.33 | 0.38 | 0.46 | 0.68 | 0.55 |
| KLHL9 | 0.33 | 0.38 | 0.33 | 0.56 | 0.38 |
| TRIM62 | 0.33 | 0.37 | 0.56 | 0.51 | 0.42 |
| FYTTD1 | 0.33 | 0.37 | 0.47 | 0.76 | 0.42 |
| SLC36A1 | 0.33 | 0.37 | 0.46 | 0.57 | 0.5 |
| ENTPD4 | 0.33 | 0.37 | 0.4 | 0.69 | 0.31 |
| CCDC115 | 0.33 | 0.37 | 0.39 | 0.43 | 0.44 |
| TMEM9B | 0.33 | 0.36 | 0.5 | 0.46 | 0.37 |
| ZBED5 | 0.33 | 0.36 | 0.49 | 0.54 | 0.51 |
| PITPNM1 | 0.33 | 0.36 | 0.42 | 0.43 | 0.36 |
| C3orf17 | 0.33 | 0.36 | 0.41 | 0.66 | 0.58 |
| TTC14 | 0.33 | 0.36 | 0.37 | 0.56 | 0.36 |
| DAZAP2 | 0.33 | 0.35 | 0.46 | 0.7 | 0.44 |
| ZC3H7A | 0.33 | 0.35 | 0.45 | 0.65 | 0.44 |
| IRF5 | 0.33 | 0.35 | 0.41 | 0.46 | 0.32 |
| ZNF410 | 0.33 | 0.34 | 0.48 | 0.61 | 0.54 |
| AEBP2 | 0.33 | 0.34 | 0.38 | 0.68 | 0.52 |
| UBE2Q2 | 0.33 | 0.34 | 0.31 | 0.46 | 0.5 |
| RNF4 | 0.33 | 0.33 | 0.52 | 0.73 | 0.55 |
| RBM7 | 0.33 | 0.33 | 0.43 | 0.62 | 0.42 |
| C7orf43 | 0.33 | 0.33 | 0.36 | 0.38 | 0.3 |
| CAPZA1 | 0.33 | 0.32 | 0.51 | 0.74 | 0.52 |
| TOP1 | 0.33 | 0.32 | 0.47 | 0.79 | 0.48 |
| TRA2B | 0.33 | 0.32 | 0.47 | 0.7 | 0.62 |
| AQR | 0.33 | 0.32 | 0.45 | 0.71 | 0.44 |
| C11orf30 | 0.33 | 0.32 | 0.44 | 0.72 | 0.4 |
| SENP6 | 0.33 | 0.32 | 0.35 | 0.67 | 0.42 |
| ZNF333 | 0.33 | 0.32 | 0.3 | 0.5 | 0.41 |
| SFRS7 | 0.33 | 0.31 | 0.5 | 0.61 | 0.62 |
| G2E3 | 0.33 | 0.31 | 0.42 | 0.67 | 0.45 |
| GNA13 | 0.33 | 0.31 | 0.4 | 0.74 | 0.44 |
| NDNL2 | 0.33 | 0.31 | 0.4 | 0.59 | 0.39 |
| SMNDC1 | 0.33 | 0.31 | 0.34 | 0.69 | 0.55 |
| ABI1 | 0.33 | 0.3 | 0.44 | 0.65 | 0.47 |
| TRIP4 | 0.33 | 0.3 | 0.44 | 0.61 | 0.62 |
| TMEM170A | 0.33 | 0.3 | 0.35 | 0.64 | 0.36 |
| ZNF286A | 0.33 | 0.3 | 0.33 | 0.55 | 0.6 |
| CYTH3 | 0.32 | 0.73 | 0.44 | 0.42 | 0.3 |
| CAV1 | 0.32 | 0.55 | 0.36 | 0.36 | 0.41 |
| LHFPL2 | 0.32 | 0.51 | 0.43 | 0.49 | 0.45 |
| AP3M2 | 0.32 | 0.5 | 0.33 | 0.51 | 0.5 |
| FAM126A | 0.32 | 0.47 | 0.36 | 0.6 | 0.31 |
| ASAP1 | 0.32 | 0.46 | 0.39 | 0.55 | 0.47 |
| GTDC1 | 0.32 | 0.45 | 0.38 | 0.54 | 0.35 |
| ABCC5 | 0.32 | 0.45 | 0.37 | 0.5 | 0.53 |
| ZSWIM6 | 0.32 | 0.45 | 0.36 | 0.54 | 0.32 |
| FAM118B | 0.32 | 0.44 | 0.47 | 0.6 | 0.56 |
| CAPZB | 0.32 | 0.44 | 0.47 | 0.45 | 0.54 |
| ATP6AP2 | 0.32 | 0.44 | 0.38 | 0.52 | 0.47 |
| STX12 | 0.32 | 0.43 | 0.5 | 0.73 | 0.38 |
| ARFIP1 | 0.32 | 0.43 | 0.45 | 0.58 | 0.32 |
| PLEKHM1P | 0.32 | 0.43 | 0.36 | 0.53 | 0.35 |
| PDCL | 0.32 | 0.4 | 0.46 | 0.62 | 0.59 |
| NPTN | 0.32 | 0.4 | 0.39 | 0.55 | 0.43 |
| NCK1 | 0.32 | 0.38 | 0.46 | 0.69 | 0.36 |
| E2F4 | 0.32 | 0.38 | 0.43 | 0.52 | 0.62 |
| KIAA0494 | 0.32 | 0.37 | 0.49 | 0.76 | 0.37 |
| BRPF1 | 0.32 | 0.37 | 0.48 | 0.66 | 0.56 |
| TRAF6 | 0.32 | 0.37 | 0.47 | 0.71 | 0.4 |
| PAPSS1 | 0.32 | 0.37 | 0.45 | 0.55 | 0.56 |
| LIMK1 | 0.32 | 0.37 | 0.42 | 0.37 | 0.51 |
| USPL1 | 0.32 | 0.37 | 0.36 | 0.55 | 0.36 |
| COG6 | 0.32 | 0.37 | 0.31 | 0.64 | 0.34 |
| MAPKBP1 | 0.32 | 0.36 | 0.47 | 0.61 | 0.5 |
| RP2 | 0.32 | 0.35 | 0.44 | 0.74 | 0.32 |
| PRKCD | 0.32 | 0.35 | 0.41 | 0.44 | 0.49 |
| EXOC5 | 0.32 | 0.35 | 0.39 | 0.69 | 0.38 |
| RNF19B | 0.32 | 0.34 | 0.61 | 0.62 | 0.44 |
| SNX2 | 0.32 | 0.34 | 0.49 | 0.68 | 0.4 |
| FAM179B | 0.32 | 0.34 | 0.31 | 0.62 | 0.36 |
| BAZ2A | 0.32 | 0.33 | 0.43 | 0.71 | 0.51 |
| TCF12 | 0.32 | 0.33 | 0.38 | 0.63 | 0.47 |
| MOSPD2 | 0.32 | 0.33 | 0.37 | 0.74 | 0.37 |
| RBM5 | 0.32 | 0.33 | 0.35 | 0.49 | 0.44 |
| LOC100271836 | 0.32 | 0.33 | 0.31 | 0.65 | 0.35 |
| EIF4G2 | 0.32 | 0.32 | 0.52 | 0.76 | 0.55 |
| TMEM102 | 0.32 | 0.32 | 0.41 | 0.32 | 0.38 |
| COPS4 | 0.32 | 0.32 | 0.4 | 0.54 | 0.49 |
| GOPC | 0.32 | 0.32 | 0.33 | 0.67 | 0.4 |
| CHSY1 | 0.32 | 0.32 | 0.3 | 0.32 | 0.41 |
| PIK3CA | 0.32 | 0.31 | 0.44 | 0.72 | 0.42 |
| POLR2B | 0.32 | 0.31 | 0.42 | 0.66 | 0.56 |
| NR2C2 | 0.32 | 0.31 | 0.39 | 0.72 | 0.4 |
| DR1 | 0.32 | 0.3 | 0.49 | 0.72 | 0.49 |
| DBR1 | 0.32 | 0.3 | 0.46 | 0.67 | 0.59 |
| SP1 | 0.32 | 0.3 | 0.42 | 0.72 | 0.49 |
| MCPH1 | 0.32 | 0.3 | 0.39 | 0.69 | 0.4 |
| TES | 0.32 | 0.3 | 0.39 | 0.43 | 0.45 |
| GPATCH8 | 0.32 | 0.3 | 0.37 | 0.69 | 0.46 |
| USP25 | 0.32 | 0.3 | 0.35 | 0.63 | 0.42 |
| EIF2AK3 | 0.32 | 0.3 | 0.34 | 0.67 | 0.35 |
| ZEB1 | 0.32 | 0.3 | 0.3 | 0.65 | 0.31 |
| SEMA4C | 0.32 | 0.3 | 0.3 | 0.46 | 0.51 |
| SEPN1 | 0.31 | 0.76 | 0.38 | 0.45 | 0.39 |
| ACER3 | 0.31 | 0.63 | 0.39 | 0.54 | 0.33 |
| RIN2 | 0.31 | 0.56 | 0.46 | 0.42 | 0.43 |
| CLIC4 | 0.31 | 0.52 | 0.43 | 0.58 | 0.31 |
| TBC1D1 | 0.31 | 0.52 | 0.37 | 0.45 | 0.35 |
| CSRNP2 | 0.31 | 0.48 | 0.32 | 0.58 | 0.43 |
| UNC93B1 | 0.31 | 0.47 | 0.4 | 0.3 | 0.44 |
| SNW1 | 0.31 | 0.46 | 0.39 | 0.54 | 0.59 |
| PRPS2 | 0.31 | 0.46 | 0.35 | 0.47 | 0.32 |
| RAB12 | 0.31 | 0.45 | 0.5 | 0.64 | 0.37 |
| CYFIP1 | 0.31 | 0.45 | 0.46 | 0.61 | 0.45 |
| SH3GLB1 | 0.31 | 0.45 | 0.42 | 0.66 | 0.48 |
| BICD1 | 0.31 | 0.45 | 0.39 | 0.61 | 0.48 |
| C20orf194 | 0.31 | 0.45 | 0.36 | 0.5 | 0.45 |
| PTPN18 | 0.31 | 0.43 | 0.32 | 0.31 | 0.35 |
| MYH9 | 0.31 | 0.42 | 0.43 | 0.59 | 0.56 |
| KIAA1632 | 0.31 | 0.42 | 0.4 | 0.68 | 0.39 |
| RNF41 | 0.31 | 0.41 | 0.41 | 0.7 | 0.54 |
| PRNP | 0.31 | 0.41 | 0.36 | 0.41 | 0.42 |
| FAM116A | 0.31 | 0.4 | 0.45 | 0.73 | 0.42 |
| FGFR1 | 0.31 | 0.4 | 0.31 | 0.32 | 0.42 |
| CFL1 | 0.31 | 0.39 | 0.49 | 0.42 | 0.6 |
| SLC7A6OS | 0.31 | 0.39 | 0.45 | 0.6 | 0.54 |
| RAB28 | 0.31 | 0.39 | 0.4 | 0.57 | 0.53 |
| ACTR10 | 0.31 | 0.39 | 0.38 | 0.53 | 0.42 |
| DNAJC10 | 0.31 | 0.39 | 0.33 | 0.55 | 0.42 |
| DULLARD | 0.31 | 0.38 | 0.33 | 0.3 | 0.53 |
| PHF2 | 0.31 | 0.38 | 0.31 | 0.58 | 0.39 |
| QKI | 0.31 | 0.37 | 0.4 | 0.66 | 0.39 |
| TFCP2 | 0.31 | 0.37 | 0.37 | 0.62 | 0.52 |
| PJA1 | 0.31 | 0.36 | 0.51 | 0.61 | 0.53 |
| SCAMP2 | 0.31 | 0.36 | 0.41 | 0.46 | 0.46 |
| ROD1 | 0.31 | 0.36 | 0.4 | 0.67 | 0.54 |
| NDE1 | 0.31 | 0.35 | 0.5 | 0.64 | 0.5 |
| SFRS13A | 0.31 | 0.35 | 0.46 | 0.75 | 0.47 |
| PRR12 | 0.31 | 0.35 | 0.38 | 0.56 | 0.5 |
| NPHP3 | 0.31 | 0.35 | 0.33 | 0.61 | 0.38 |
| ISY1 | 0.31 | 0.34 | 0.47 | 0.55 | 0.71 |
| FCF1 | 0.31 | 0.34 | 0.44 | 0.65 | 0.51 |
| FBXL14 | 0.31 | 0.34 | 0.39 | 0.54 | 0.34 |
| ARPP19 | 0.31 | 0.34 | 0.37 | 0.65 | 0.43 |
| RBM26 | 0.31 | 0.34 | 0.33 | 0.62 | 0.43 |
| BAG5 | 0.31 | 0.33 | 0.45 | 0.65 | 0.43 |
| MRE11A | 0.31 | 0.33 | 0.44 | 0.7 | 0.55 |
| PRDM4 | 0.31 | 0.33 | 0.43 | 0.64 | 0.59 |
| EFHD2 | 0.31 | 0.33 | 0.42 | 0.32 | 0.44 |
| PCNP | 0.31 | 0.33 | 0.4 | 0.76 | 0.56 |
| RBM38 | 0.31 | 0.33 | 0.32 | 0.33 | 0.44 |
| RASA1 | 0.31 | 0.32 | 0.41 | 0.69 | 0.36 |
| WTAP | 0.31 | 0.32 | 0.41 | 0.65 | 0.47 |
| ZC3H4 | 0.31 | 0.32 | 0.4 | 0.59 | 0.49 |
| ZNF224 | 0.31 | 0.32 | 0.33 | 0.51 | 0.33 |
| VRK2 | 0.31 | 0.31 | 0.49 | 0.74 | 0.5 |
| C18orf25 | 0.31 | 0.31 | 0.48 | 0.77 | 0.47 |
| ELF2 | 0.31 | 0.31 | 0.39 | 0.6 | 0.44 |
| RBM16 | 0.31 | 0.31 | 0.36 | 0.7 | 0.42 |
| RBM45 | 0.31 | 0.3 | 0.51 | 0.69 | 0.54 |
| IWS1 | 0.31 | 0.3 | 0.43 | 0.63 | 0.56 |
| ATG4C | 0.31 | 0.3 | 0.42 | 0.65 | 0.36 |
| GTPBP1 | 0.31 | 0.3 | 0.42 | 0.43 | 0.52 |
| PTMA | 0.31 | 0.3 | 0.41 | 0.44 | 0.55 |
| PDCD7 | 0.31 | 0.3 | 0.37 | 0.6 | 0.55 |
| RALGAPB | 0.31 | 0.3 | 0.36 | 0.68 | 0.51 |
| POC1B | 0.31 | 0.3 | 0.31 | 0.69 | 0.38 |
| MYOF | 0.3 | 0.54 | 0.3 | 0.33 | 0.33 |
| MPRIP | 0.3 | 0.52 | 0.33 | 0.3 | 0.33 |
| SLC9A1 | 0.3 | 0.52 | 0.31 | 0.32 | 0.36 |
| RNF6 | 0.3 | 0.5 | 0.39 | 0.68 | 0.32 |
| TMEM43 | 0.3 | 0.49 | 0.4 | 0.45 | 0.5 |
| RHOQ | 0.3 | 0.49 | 0.32 | 0.45 | 0.34 |
| DYNC1LI2 | 0.3 | 0.48 | 0.41 | 0.63 | 0.45 |
| OSBPL3 | 0.3 | 0.48 | 0.35 | 0.41 | 0.41 |
| WWC3 | 0.3 | 0.47 | 0.44 | 0.52 | 0.42 |
| ZNF436 | 0.3 | 0.47 | 0.41 | 0.59 | 0.37 |
| GPR176 | 0.3 | 0.46 | 0.48 | 0.46 | 0.32 |
| VPS24 | 0.3 | 0.46 | 0.36 | 0.5 | 0.5 |
| KIDINS220 | 0.3 | 0.45 | 0.38 | 0.74 | 0.38 |
| IFT57 | 0.3 | 0.44 | 0.44 | 0.44 | 0.57 |
| BRMS1L | 0.3 | 0.44 | 0.43 | 0.65 | 0.45 |
| MTMR2 | 0.3 | 0.44 | 0.41 | 0.54 | 0.57 |
| RHOA | 0.3 | 0.43 | 0.45 | 0.63 | 0.54 |
| MSRB3 | 0.3 | 0.43 | 0.36 | 0.44 | 0.3 |
| MARK3 | 0.3 | 0.42 | 0.41 | 0.49 | 0.51 |
| PPP3CA | 0.3 | 0.42 | 0.35 | 0.49 | 0.34 |
| ATMIN | 0.3 | 0.41 | 0.45 | 0.57 | 0.37 |
| TP53BP1 | 0.3 | 0.41 | 0.45 | 0.56 | 0.55 |
| YEATS2 | 0.3 | 0.4 | 0.47 | 0.62 | 0.6 |
| CDYL | 0.3 | 0.4 | 0.42 | 0.61 | 0.56 |
| CTTNBP2NL | 0.3 | 0.4 | 0.39 | 0.48 | 0.36 |
| KDELC2 | 0.3 | 0.39 | 0.49 | 0.68 | 0.39 |
| USP24 | 0.3 | 0.39 | 0.46 | 0.7 | 0.43 |
| SLC25A24 | 0.3 | 0.38 | 0.43 | 0.49 | 0.32 |
| APPL1 | 0.3 | 0.38 | 0.39 | 0.75 | 0.41 |
| DNAJC13 | 0.3 | 0.37 | 0.45 | 0.74 | 0.49 |
| GIGYF2 | 0.3 | 0.37 | 0.38 | 0.65 | 0.49 |
| EXT2 | 0.3 | 0.36 | 0.52 | 0.57 | 0.56 |
| ARF3 | 0.3 | 0.36 | 0.44 | 0.63 | 0.53 |
| LRP10 | 0.3 | 0.36 | 0.43 | 0.43 | 0.37 |
| TMEM128 | 0.3 | 0.36 | 0.37 | 0.53 | 0.41 |
| BCL9L | 0.3 | 0.36 | 0.36 | 0.53 | 0.47 |
| BRD1 | 0.3 | 0.36 | 0.32 | 0.54 | 0.56 |
| RABGAP1 | 0.3 | 0.36 | 0.3 | 0.58 | 0.47 |
| UBE2E1 | 0.3 | 0.35 | 0.43 | 0.58 | 0.61 |
| KIAA0831 | 0.3 | 0.35 | 0.31 | 0.54 | 0.39 |
| PWWP2A | 0.3 | 0.34 | 0.44 | 0.69 | 0.4 |
| CEP170 | 0.3 | 0.34 | 0.34 | 0.63 | 0.44 |
| RAB11A | 0.3 | 0.34 | 0.34 | 0.54 | 0.51 |
| ZNF304 | 0.3 | 0.34 | 0.33 | 0.55 | 0.44 |
| ZNF646 | 0.3 | 0.33 | 0.48 | 0.64 | 0.49 |
| IL17RA | 0.3 | 0.33 | 0.46 | 0.51 | 0.56 |
| GNL3L | 0.3 | 0.33 | 0.42 | 0.66 | 0.53 |
| KCTD7 | 0.3 | 0.33 | 0.35 | 0.61 | 0.5 |
| GLTP | 0.3 | 0.32 | 0.46 | 0.54 | 0.49 |
| PHF21A | 0.3 | 0.32 | 0.45 | 0.61 | 0.52 |
| NCOA1 | 0.3 | 0.32 | 0.44 | 0.64 | 0.39 |
| LASS5 | 0.3 | 0.32 | 0.39 | 0.48 | 0.64 |
| MARCKS | 0.3 | 0.32 | 0.39 | 0.45 | 0.59 |
| REV1 | 0.3 | 0.32 | 0.38 | 0.66 | 0.48 |
| SACM1L | 0.3 | 0.32 | 0.32 | 0.67 | 0.3 |
| MADD | 0.3 | 0.31 | 0.45 | 0.59 | 0.51 |
| WHSC1L1 | 0.3 | 0.31 | 0.43 | 0.67 | 0.47 |
| FBXO11 | 0.3 | 0.31 | 0.41 | 0.72 | 0.48 |
| GMFB | 0.3 | 0.31 | 0.41 | 0.67 | 0.44 |
| ERCC3 | 0.3 | 0.31 | 0.41 | 0.64 | 0.6 |
| TTF1 | 0.3 | 0.31 | 0.41 | 0.51 | 0.57 |
| CLASP1 | 0.3 | 0.31 | 0.4 | 0.66 | 0.41 |
| TOP2B | 0.3 | 0.31 | 0.39 | 0.59 | 0.58 |
| ZBTB26 | 0.3 | 0.31 | 0.37 | 0.62 | 0.42 |
| USP11 | 0.3 | 0.31 | 0.37 | 0.43 | 0.56 |
| ZNF567 | 0.3 | 0.31 | 0.32 | 0.62 | 0.48 |
| NOL8 | 0.3 | 0.31 | 0.3 | 0.56 | 0.52 |
| PHIP | 0.3 | 0.31 | 0.3 | 0.55 | 0.49 |
| IQCB1 | 0.3 | 0.3 | 0.44 | 0.65 | 0.57 |
| SF1 | 0.3 | 0.3 | 0.44 | 0.6 | 0.63 |
| RTF1 | 0.3 | 0.3 | 0.4 | 0.67 | 0.45 |
| DCAF16 | 0.3 | 0.3 | 0.34 | 0.51 | 0.54 |
| POLG | 0.3 | 0.3 | 0.34 | 0.49 | 0.36 |
| STXBP5 | 0.3 | 0.3 | 0.3 | 0.55 | 0.38 |
